# Discrete distributions are learnable from metastable samples

**DOI:** 10.1038/s41467-026-75439-1

**Published:** 2026-07-23

**Authors:** Abhijith Jayakumar, Andrey Y. Lokhov, Sidhant Misra, Marc Vuffray

**Affiliations:** 1 Theoretical Division, Los Alamos National Laboratory, Los Alamos, NM USA; 2 Center for Quantum Computing, Los Alamos National Laboratory, Los Alamos, NM USA

**Keywords:** Applied mathematics, Statistical physics

## Abstract

Physically motivated stochastic dynamics are widely used to sample from high-dimensional distributions. However, such samplers often get trapped in metastable states, approximately sampling from a distribution that differs significantly from the desired stationary state. We rigorously show that for multivariable discrete distributions, the true stationary model can nevertheless be recovered from these metastable samples. This relies on a fundamental observation: for distributions satisfying a strong metastability condition, their single-variable conditional probabilities are on average extremely close to those of the true stationary distribution. This remains true even when the two distributions are far apart under global metrics such as Kullback-Leibler divergence. Consequently, we can effectively learn the true model using a conditional-likelihood estimator even when the samples are drawn from a restricted state space. Extending these general results to Ising models, we prove rigorous parameter and structure learning guarantees. Finally, we demonstrate this phenomenon numerically on higher-alphabet spin glass models.

## Abstract (web summary)

Algorithms sampling from thermal states of many-body systems often get stuck in restricted, metastable states. Here, authors prove that the true underlying models can nevertheless be accurately recovered from these trapped samples, establishing rigorous learning guarantees for discrete distributions.

## Introduction

Markov chains are by far the most popular tool used to study systems described by many-variable probability distributions. It is well known that Markov chains can mix very slowly to the stationary distribution, and this is often associated with the existence of so-called metastable states, where the chain can get stuck for long periods of time^1–4^. Slow mixing in such systems is not merely an algorithmic inconvenience but is a pervasive property observed in natural systems from molecular biology to quantum field theory^5–8^. Many recent works that give rigorous guarantees on learning such distributions work under the assumption that independent and identically distributed (i.i.d.) samples are available from the ground truth distribution^9–12^. This assumption is well justified in contexts where data is collected from independent agents in a real-world setting. However, it is less likely to hold in cases where the source of the data is a natural dynamical system or Markov chain sampling algorithm, due to slow mixing often exhibited by such systems^13–15^. These observations raise the following question: Is it possible to learn something useful about the stationary distribution of a Markov chain given samples drawn from such a metastable state of the chain?

Prima facie, this task looks hopeless. A chain exhibiting metastability often samples from a restricted part of a state space. This, in turn, implies that metastable distributions will be quite different from the stationary distribution when their difference is measured using global metrics like Kullback–Leibler (KL) divergence or total variation (TV) distance. This can have severe consequences for learning using certain algorithms that attempt to minimize global metrics between the data distribution and a hypothesis. For instance, the maximum likelihood estimator (MLE) implicitly attempts to minimize the KL divergence. Due to the large KL divergence between the metastable and stationary distributions, the MLE estimate will never approximate the true distribution well, even if the MLE uses a large amount of data from the metastable distribution.

However, for the purposes of learning, it is not always necessary or useful to minimize such global metrics. For example, consider the problem of learning undirected graphical models. Efficient algorithms for this problem, like pseudo-likelihood (PL)^16–18^, interaction screening^19,20^, and Sparsitron^21^, work by learning the single-variable conditionals of this distribution (i.e., the conditional distributions of one variable where other variables are fixed). These works exploit the fact that there is a bijective mapping between positive distributions over discrete variables and their single-variable conditionals^22^. It might then seem that samples from the true distribution are necessary to even approximately learn the conditionals. In this work, we will establish that this is not the case. That is, there exist other distributions that can be globally different from the true distribution but have conditionals that are, on average, very close to the ground-truth single-variable conditionals. We will explicitly show that metastable distributions of reversible Markov chains that have the true model as its equilibrium distribution satisfy such a property.

The main theoretical motivation for studying learning from metastable distributions comes from the observation that many instances of multivariable discrete distributions are believed to be hard to sample from^13,23,24^. This necessarily leads to poor mixing of Markov chain samplers, which is usually observed in the low-temperature region, when the variables in the model interact strongly^4,25^. Now, from the context of learning, it is natural to ask if something about the stationary distribution can be reconstructed from data produced by such a Markov chain.

For the purposes of learning, we will model the stationary distribution as a Gibbs distribution with a certain energy function, equivalently as an undirected graphical model^26^. The theory of learning undirected graphical models with discrete variables in the setting where i.i.d. samples are given from the true distribution is well developed^17–21,27,28^. Recent works have established that methods that learn the parameters of the model by minimizing metrics based on single-variable conditionals achieve efficient sample and computational complexity scaling. Some works have also demonstrated efficient learning in the setting where samples are given as a time series from a Markov chain sampler^29–32^. Notice that this setting is markedly different from learning from metastable distributions. In our case, we do not observe any dynamical information from the Markov chain. Instead, we assume that the samples we have are i.i.d. from a metastable distribution of the Markov chain.

An alternative perspective on Markov chains and local learning methods has been explored in some recent works in the context of learning energy-based models with continuous variables^33,34^. These works show that such models that do not bottleneck the probability flow are efficiently learnable by a local learning method known as score matching. In our work, in the discrete variable setting, we show that the presence of large bottlenecks does not impede learning.

Metastability in the context of multivariable discrete systems has been well explored in the statistical physics literature^1,35,36^ due to the connection between such distributions and the emergence of different thermodynamic phases in models of interacting systems. A rigorous examination of this phenomenon has also been undertaken by some authors^4,24^ who show specific examples of regions within which stochastic dynamics can mix well, but the chain struggles to escape the said region effectively. More recently, Liu et al.^37^ have also explored metastability in Markov chains and have demonstrated rigorous algorithmic utility for data produced from these states.

Informally, the primary result of our work can be stated as follows: Let $\mu (\underline{\sigma })$ be a distribution over a set of discrete variables and let *P* be a Markov chain that has *μ* as its equilibrium distribution; we define a family of metastable distributions of *P* such that given i.i.d. samples from these distributions, the stationary distribution *μ* can be learned to near optimal accuracy using the pseudo-likelihood method. This family of metastable distributions is defined by an approximate detailed balance condition. Moreover, metastable distributions that represent a chain stuck in a region of state space can be shown to lie in this family.

The rest of the paper is dedicated to defining metastable distributions, establishing these definitions by examples, and then using the notion of metastability to give learning guarantees. This leads to the discovery of elegant connections between several ideas from statistical physics and learning theory.

We begin in Section “Definitions of Metastability,” by defining two notions of metastability by relaxing, respectively, the stationarity and detailed-balance conditions that are obeyed by the equilibrium distribution of a reversible Markov chain (*P*). The general definition of metastability corresponds to distributions that only change by *η* in the total variation distance (TV) when updated by the Markov chain. It follows that when *η* = 0 this condition is satisfied only by the equilibrium distribution. This definition of metastability captures the intuitive notion of a metastable distribution as one that does not change much with time. We then define a second notion of metastability, which we can call *η*-*strong metastability*. These are defined as distributions that violate the detailed balance condition of *P* by *η*. Here, the violation is again measured in TV. Just like how the exact detailed balance condition of a distribution with respect to *P* implies that it is a stationary state of *P*, the strong notion of metastability can be trivially shown to imply the notion of metastability. To foreshadow the learning results, we will later show that samples from strongly metastable states allow us to recover the true energy function describing the stationary distribution of *P*.

The question remains whether there are interesting or relevant cases of strong metastable distributions. We show two explicit constructions of strongly metastable distributions that show these states do exist and that they align with the intuitive notion of a metastable system as one that is stuck in a region of state space, or as a system trapped in local minima of free energy^38,39^. For general reversible Markov chains, we construct *η*-strongly metastable states by using the notion of conductance from the theory of Markov chains. We show that any subset in the state space supports a *η*-strong metastable state with *η* proportional to the conductance of the subset. For the case of slow mixing chains, this implies that there exists a *η* strongly metastable state with *η* being exponentially small in the number of variables in the system. Moreover, the explicit constructions we have correspond to distributions resulting from Markov chains that have mixed well inside a set but have no support outside of it, essentially being stuck in such a set. In the final section of the paper, we illustrate another class of strongly metastable states in the Curie–Weiss (CW) model. These distributions are peaked around the minima of the free energy of the system and can also be shown to have an *η* that decays with the number of variables in the model.

After establishing the notion of strong metastability, we move on to showing the connection between strongly metastable distributions and learning discrete distributions. The key connection we show is that strongly metastable states have, on average, almost the same single-variable conditionals as the equilibrium distribution *μ*, which is the true distribution we are ultimately interested in learning.

For our learning setup, we model our distributions as Gibbs distributions with some energy function ($\mu (\underline{\sigma })\propto {e}^{{E}^{* }(\underline{\sigma })}$) and aim to learn this energy function by optimizing over a parametric family of energy functions using the pseudo-likelihood method. Now, given some natural assumptions about the learning problem (see Conditions 1 and 2), we rigorously establish that strongly metastable states are almost as good as the equilibrium distribution for learning the energy function. Below, we informally state the main result that facilitates this.

Theorem 1 - closeness of conditionals: The single-variable conditionals of an *η*-strongly metastable state of *P* differ from those of the equilibrium distribution *μ* in average TV distance by only *O*(*η*). The average in this case is taken over the conditioning variables using the metastable distribution.

**Fig. 1:**
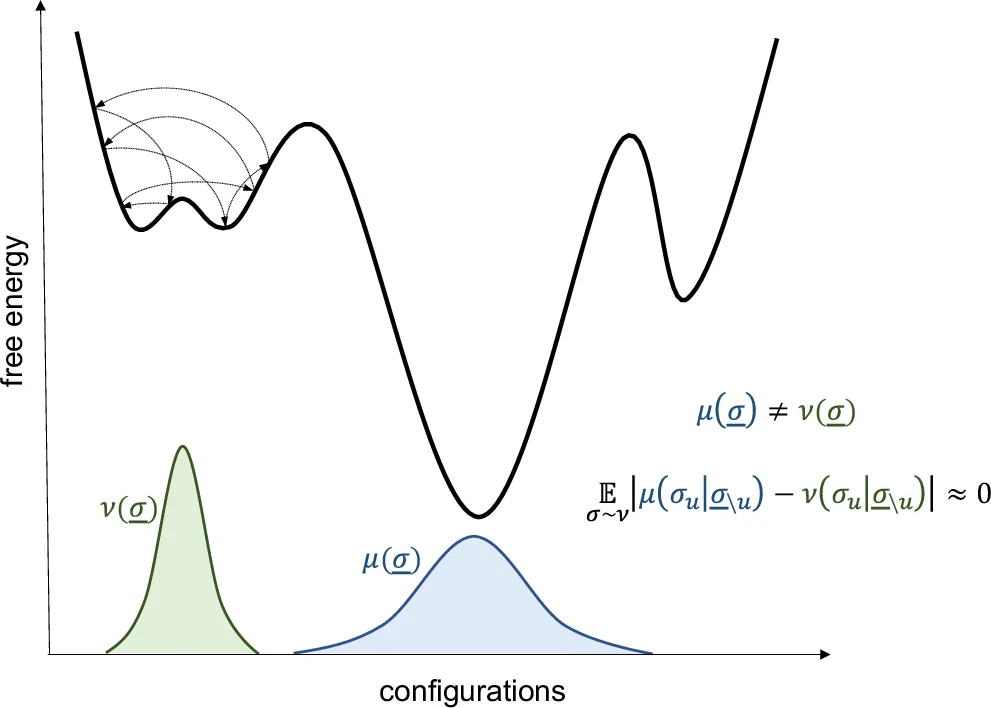
An informal representation of our result given by Theorem 1. Samples coming from a metastable distribution of reversible Markov chain samplers are far from the full measure in global metrics. Surprisingly, at the same time, we show that single-variable conditionals in metastable distributions are, on average, close to those of the true distribution. In the Curie–Weiss model, we will use an explicit construction to demonstrate that such metastable states correspond to the local minima of the free energy, which agrees with an intuitive statistical physics picture of metastability.

This is schematically represented in Fig. 1. This result is crucial for learning as the PL method works by minimizing the average KL divergence between single-variable conditionals. Specifically, given samples from the data distribution and a parametric family of energy functions, the PL method estimates the energy function from the data by minimizing the average KL divergence between the single-variable conditionals of the data and the model, averaged over the data distribution^16,22^. The data distribution in our case is given by the i.i.d. samples drawn from a strongly metastable distribution. Now, using the well-known bounds connecting TV to the KL divergence, we can argue that the PL loss computed from samples drawn from a strongly metastable distribution is close to its optimal value when the parameters of the model hypothesis match that of the stationary distribution of *P*.

Notice from our constructions that *η* for strong metastable states is often a decaying function of the number of variables for slow-mixing systems. This implies that for large systems that exhibit slow mixing, the *O*(*η*) bias incurred in the PL loss function is dominated by the statistical errors that scale as *M*^−1/2^. These bounds on the PL loss computed from metastable samples are given formally in Theorem 2.

These “nearly optimal” results can be further developed into parameter learning guarantees. We demonstrate this for the special case of binary undirected graphical models with pairwise interactions. In other words, we solve the inverse Ising problem given data from metastable states of the model^40,41^.

We use tools from statistical learning theory to show that the parameters of the energy function of pairwise binary (Ising) exponential family models can be reconstructed from samples from metastable states. We show that the coupling and magnetic field parameters of such models can be learned using ${e}^{O(\beta d)}\log (n)$ samples for an *n*-spin system with only a bias that scales with *η*. Hence, in the most interesting cases (i.e., like in Section “Existence of Strongly Metastable Distributions for Reversible Markov Chains” for a slow mixing chain), this bias is exponentially small in the number of spins and is completely dominated by the statistical error in the learning process.

In the final section of the paper, we give a numerical illustration of our results using the CW ferromagnet and a spin glass model with higher-order interactions. By exploiting the permutation symmetry of the CW model, we numerically show that there are strongly metastable states supported around the minima of the free energy of this model. We also collect metastable data from this model by running Glauber dynamics around one of the free energy minima and show that the full model parameters can be learned faithfully from this data using the PL method. For the spin glass model, we demonstrate learning from metastable distributions on a spin glass model with higher-order interactions and a three-level alphabet.

## Results

### Definitions of metastability

A metastable distribution must be a distribution that behaves similarly to the stationary distribution under a time update. From this, a natural definition of a metastable distribution can be given by bounding the change in the distribution after one step of a Markov chain.

#### Definition 1

(Metastability) A distribution *ν* is *η*- metastable with respect to a Markov chain *P* if and only if,

1 $$| \nu \,-\,\nu P{| }_{{{\mathrm{TV}}}}\le \eta$$

This definition most naturally captures the idea of a metastable distribution. A similar notion of metastability has been recently defined in ref. ^37^ using the KL divergence instead of TV. Later, we will show explicit constructions where *η* is smaller than some inverse polynomial function of the size of the state space of the chain. In the language of high-dimensional distributions, this implies an exponentially small quantity in the number of variables. It can be shown that if the Markov chain is started from such a distribution, then that chain will mix poorly to the stationary distribution.

#### Proposition 1

Starting from an *η*-metastable distribution, it takes at least $\frac{| \mu -\nu {| }_{TV}-\epsilon }{\eta }$ steps to get *ϵ* close to the equilibrium distribution *μ* in TV.

This simple fact is a consequence of the data-processing inequality. The proof is given in Supplementary Note 1.

However, almost all Markov chain samplers used in practice have a stationary state that satisfies time reversibility, which is captured by the detailed balance condition. For reversible Markov chains, the detailed balance condition can be shown to imply the existence of a stationary distribution. For a distribution *μ* and a chain *P* with a state space ${{{\mathcal{S}}}}$, the following implication holds true,

2 $$P(i|\,\, j)\mu (\,\,j)=P(\,\,j| i)\mu (i),\,\forall i,j\in {{{\mathcal{S}}}}\ \Rightarrow \ \mu P=\mu$$

Now, if we take the view that this time reversibility condition must only be mildly violated for a metastable distribution, then a stronger notion of metastability emerges.

#### Definition 2

(Strong metastability) Let *P* be a Markov chain with a state space ${{{\mathcal{S}}}}$. Then a distribution *ν* is *η*-strongly metastable with respect to *P* iff,

3 $$\frac{1}{2}{\sum}_{i,j\in {{{\mathcal{S}}}}}| P(i| \,\,j)\nu (\,\,j)-P(\,\,j| i)\nu (i)| \,\le \,\eta$$

If we set *η* to zero in the definition of strong metastability, then we get the detailed balance condition, *P*(*i*∣ *j*)*ν*( *j*) = *P*( *j*∣*i*)*ν*(*i*). As foreshadowed by their names, it can be easily deduced from their definitions that strong metastability implies metastability with the same *η*.

In the following sections, we will show explicit constructions of strongly metastable distributions that apply to general reversible chains. We will then use this definition of strong metastability to show how the equilibrium distribution can be learned given samples drawn from such a state. We also discuss more closely the relationship between these two notions of metastability in Supplementary Note 2. Specifically, in Proposition 4, we show that it is possible to have large separations between these two measures of metastability, implying that the strong metastability condition is indeed the relevant condition to look for in the context of learning.

### Existence of strongly metastable distributions for reversible Markov chains

From the definition of strong metastability, it is not clear whether metastable distributions seen while sampling from challenging many-variable problems satisfy this condition. We will show that strong metastability is closely tied to regions in the state space that bottleneck the probability flow. This will further imply that any slow-mixing reversible Markov chain must necessarily have strongly metastable distributions.

To this end, let us first define *conductance*, which captures the ease with which probability can flow out of a certain region in phase space.

#### Definition 3

(Conductance) Given a Markov chain *P* on a state space ${{{\mathcal{S}}}}$ with a stationary state *μ*. The conductance of any set $A\subseteq {{{\mathcal{S}}}}$,

4 $$\Gamma (A):=\frac{{\sum}_{j\in A,i\in {A}^{c}}P(i| \,\,j)\mu (\,\,j)}{{\sum}_{j\in A}\mu (\,\,j)}$$

From this, the conductance of the chain is defined as,

5 $$\Gamma :\!\!={{\min }_{A:\mu (A) < 1/2}}\Gamma (A)$$

Conductance is a well-studied property of Markov chains and forms the basis of many classic results in randomized algorithms^42–45^. The usefulness of this property comes from the Cheeger bound, which connects it to the spectral gap of the chain and, in turn, to the mixing time.

#### Lemma 1

(Cheeger bound^42^) Let *λ*_2_ be the second-largest eigenvalue of a reversible, irreducible Markov chain *P* with conductance *Γ*. Then,

6 $$2\Gamma \ge 1-{\lambda }_{2}\ge \frac{{\Gamma }^{2}}{2}$$

Now let $1={\lambda }_{1} > {\lambda }_{2}\ge \ldots {\lambda }_{| {{{\mathcal{S}}}}| } > -1,$ be the eigenvalues of a reversible, ergodic, irreducible chain. It is well known that the mixing time of such a chain is determined by the inverse spectral gap, *τ*_*mix*_ = *Θ*(1/(1 − *λ*_2_)) ^45,46^. Now, let us look at the implications of these statements for a reversible Markov chain that is attempting to sample from a family of distributions on *n* discrete variables, each taking a value from a set *Q*. In this case ${{{\mathcal{S}}}}={Q}^{n}$. Furthermore, assume that the chain is only allowed to make moves that change at most one variable at a time. This condition is satisfied by both Glauber dynamics and the Metropolis–Hastings algorithm. Slow mixing in such a chain implies that the mixing time of the chain scales exponentially with *n*. From the Cheeger bound, this implies that there exists a set with an exponentially small conductance. We will now show that such a set will necessarily support a strongly metastable distribution.

For $A\subset {{{\mathcal{S}}}}$ define the following distribution,

7 $${\mu }_{A}(\underline{\sigma }): \!\!=\left\{\begin{array}{l}\frac{\mu (\underline{\sigma })}{\mu (A)},\,\,\underline{\sigma }\in A,\\ 0,\,\,\underline{\sigma }\in {A}^{c}.\end{array}\right.$$

Observe that the detailed balance condition is only violated at the boundary of this set. From this observation we can directly compute the strong metastability of such a state,

8 $$\frac{1}{2}{\sum}_{\underline{\sigma },{\underline{\sigma }}^{{\prime} }\in {{{\mathcal{S}}}}}| P(\underline{\sigma }| {\underline{\sigma }}^{{\prime} }){\mu }_{A}({\underline{\sigma }}^{{\prime} })-P({\underline{\sigma }}^{{\prime} }| \underline{\sigma }){\mu }_{A}(\underline{\sigma })|={\sum}_{\underline{\sigma }\in A,{\underline{\sigma }}^{{\prime} }\in {A}^{c}}| P({\underline{\sigma }}^{{\prime} }| \underline{\sigma }){\mu }_{A}(\underline{\sigma })|=\Gamma (A).$$

Thus by applying Cheeger bound to a slow-mixing reversible Markov chain *P* with stationary distribution *μ*, we conclude that there exists an *η*-strongly metastable distribution with *η* exponentially small in the number of spins.

In statistical physics, the idea of a metastable state is connected to the minima of a free energy function^1^. We also explore this connection in Section “Strongly Metastable Distributions in the Curie–Weiss Model” and demonstrate the existence of strongly metastable distributions supported around the minima of the free energy of the CW model.

### Robustness of strong metastability

To gauge the utility of the notion of strong metastability, we need to understand how it behaves when a mixture of such distributions is taken and how it behaves under dynamics. We show that the property of strong metastability is robust to both these actions.

#### Proposition 2

Let {*ν*_*k*_} be a collection of *η*_*k*_-strongly metastable distributions. For any convex combination *ν* = ∑_*k*_ *p*_*k*_*ν*_*k*_, with *p*_*k*_ ≥ 0 and ∑_*k*_ *p*_*k*_ = 1, the distribution *ν* is $\left({\sum }_{k}{p}_{k}{\eta }_{k}\right)$-strongly metastable.

This property is important because the dynamics can transition between metastable states, so the data used for learning may be drawn from mixtures of such distributions. It is also necessary to make sure that a strongly metastable distribution retains this property as it evolves according to the Markov chain. For the weaker notion of metastability, data processing inequality implies that if *ν* is *η*-metastable, then so is *νP*. We can show that an similar statement can be made for strong metastability.

#### Proposition 3

Let *ν* be an *η*-strongly metastable distribution for a reversible Markov chain *P*. Then, for every integer *t* ≥ 1, the distribution *νP*^*t*^ is (1 + 2*t*)*η*-strongly metastable.

Proofs of Propositions 2 and 3 are given in Supplementary Note 4.

### Learning from metastable distributions

Now that we have established that interesting cases of strongly metastable distributions exist, we will show that the energy function of the true distribution can be learned given samples from a strongly metastable distribution. The key result that we will exploit to learn from metastable distributions is the fact that strong metastability, when combined with the time reversibility of the chain, implies that the single-variable conditionals of the metastable distribution are close to those of the stationary state of the Markov chain. Building on this, we then use prior information on the true model and stochastic convex optimization techniques to show that learning from metastable distributions can be successfully performed using the pseudo-likelihood method.

To this end, we assume a technical condition on the transition probability of a reversible Markov chain.

#### Condition 1

(Bounded spin-flip probability) For a reversible chain *P*, let $P({\underline{\sigma }}_{u\to q}| \underline{\sigma })$ be the probability of variable at position *u* being changed to *q* ∈ *Q* when the system is in the configuration $\underline{\sigma }$. Then we assume that the ratio of this to the single-variable conditionals is lower bounded by a quantity that depends on *P*,

9 $$\frac{P({\underline{\sigma }}_{u\to q}| \underline{\sigma })}{\mu (q| {\underline{\sigma }}_{\setminus u})}\ge {\omega }_{P} > 0,\,\forall \,{\underline{\sigma }}_{\setminus u}\in {Q}^{n-1},\,q\in Q,\,u\in [n].$$

The bound in the above condition is satisfied by both Glauber dynamics and the Metropolis–Hastings sampler^47^. For Glauber dynamics, by definition of the algorithm ${\omega }_{P}=\frac{1}{n}$. For the Metropolis–Hastings Markov chain, *ω*_*P*_ will also be proportional to 1/*n*, with the ratio between them only depending on the inverse temperature of the chain.

We will later see that *ω*_*P*_ will control the error in the learning procedure, and metastable states with small *η*/*ω*_*P*_ give us good learning guarantees.

Now we state one of the main results of the paper, the proof of this result is given in the Supplementary Note 5.

#### Theorem 1

(Conditionals of strong metastable distributions) Consider a system of *n* discrete random variables where each of them can take values from the set *Q*. Let *ν* be an *η*-strongly metastable distribution of a reversible Markov chain *P* with a stationary state *μ*. If this chain satisfies Condition 1, then the single-variable conditionals of *ν* are close to those of *μ* in the following sense,

10 $${\sum}_{u=1}^{n}{\sum}_{\sigma \in {Q}^{n}}\nu (\sigma ){\left\vert \nu (.| {\underline{\sigma }}_{\setminus u})-\mu (.| {\underline{\sigma }}_{\setminus u})\right\vert }_{TV}\le \frac{\eta }{{\omega }_{P}}$$

This is a central observation in this paper, which says that a strong metastable distribution has, on average, single-variable conditionals that are very close to that of the equilibrium distribution. This result will be the basis for showing that the parameters of the true distribution (*μ*) can be learned given samples from (*ν*). This is due to the fact that methods like PL and Interaction Screening that efficiently learn discrete Gibbs distributions rely on minimizing average metrics over the single-variable conditionals.

Theorem 1 should be contrasted with the explicit constructions in Eq. 7 that gave strongly metastable distributions that are considerably far from the equilibrium in terms of global TV. According to that construction, the TV distance between the metastable distribution from the equilibrium distribution is ∣*μ*_*A*_ − *μ*∣_TV_ = 1 − *μ*(*A*), which is generally not an exponentially small quantity even if the Markov chain mixes slowly. On the other hand, the distance in Theorem 1 for *ν* = *μ*_*A*_ is *Γ*(*A*)/*ω*_*P*_, which can be exponentially small if *A* is a set that causes slow mixing in the Markov chain. We demonstrate this fact explicitly for the CW model in Section “Strongly Metastable Distributions in the Curie-Weiss Model.”

Theorem 1 implies that the metastable distribution supported on one of the minima of the free energy function has conditionals that are very close to the true distribution. This can be true even if the true model has very low mass within the support of the metastable state, as evidenced by the numerical experiments in Section “Strongly Metastable Distributions in the Curie–Weiss Model.” Consequently, learning algorithms that minimize global distance measures between distributions will not fare well for learning *μ* given data from a strongly metastable state. For instance, maximum likelihood estimation (MLE) minimizes the KL divergence between the data distribution and a proposed parametric hypothesis. The KL divergence between *μ*_*A*_ and *μ* is given by $-\log (\mu (A))$. Thus, the true model *μ* is very far from being optimal for MLE when data comes from the metastable state *μ*_*A*_. Also, the MLE can be seen as trying to match the sufficient statistics of the data to the hypothesized model^47^, and it is usually the case that sufficient statistics of metastable distributions do not match that of the stationary distribution. So, for the purpose of recovering the true model from a metastable state, we have to rely on minimizing the distance between conditionals and not global metrics.

Theorem 1 can be modified to give closeness in terms of other metrics like the average KL divergence between conditionals. This is a simple consequence of the reverse Pinsker inequality (Lemma 4.1 in ref. ^48^), which upper bounds the KL divergence between two distributions in terms of the TV. Given distributions *p* and *q*, we have, ${D}_{KL}(p| | q)\le \frac{2}{{\min }_{i}q(i)}| p-q{| }_{{{\mathrm{TV}}}}$. Using this relation directly on Theorem 1, we get,

#### Corollary 1

For distributions *μ* and *ν* as defined in Theorem 1, for every *u* ∈ [*n*] we have the following bound,

11 $${\sum}_{u=1}^{n}{{\mathbb{E}}}_{{\underline{\sigma }} \sim \nu}{D}_{{\mathrm{KL}}}(\nu (.| {\underline{\sigma }}_{\backslash u})| | \mu (.| {\underline{\sigma }}_{\backslash u}))\le \frac{2\eta }{{\omega }_{P}{\min }_{u,\underline{\sigma }}\mu ({\sigma }_{u}| {\sigma }_{\backslash u})}.$$

This metric is specifically important to bound, as the PL estimator precisely minimizes the average KL divergence between conditionals. The lower bound on the conditional is a natural quantity that controls the error in these types of learning results^16,17,21,49^. For instance, in the Sparsitron algorithm^21^, the authors use the term δ-unbiased conditionals to refer to the existence of such a lower bound.

### Test error bound for the multi-alphabet pseudo-likelihood method/logistic regression

We will now look at the consequences of the results above for learning the distribution *μ* given independent samples from an *η*-strongly metastable distribution *ν*. For this purpose, we will parametrize the ground truth distribution as a Gibbs distribution associated with an energy function, $\mu (\underline{\sigma })\propto \exp ({E}^{* }(\underline{\sigma })).$

Here, is E* is a general real-valued function on the state space *Q*^*n*^, and for strictly positive distributions, such an energy-based representation does not restrict the distribution in any way. We will be using PL to learn the true energy function associated with *μ*.

Now, for the PL algorithm, it is necessary to have a parametric hypothesis for the energy. The PL method is then essentially a regression over this parametric family to learn the true energy. Denote these parametric energy functions as $E(\underline{\sigma },\underline{\theta })$ where $\underline{\theta }$ is a set of parameters. For example, one particular choice of a parametric family could be polynomials of a certain degree over the variables $\underline{\sigma }$, in which case $\underline{\theta }$ can be chosen as the coefficients in the polynomial. Another parametrization, that has gained popularity in ML literature, is a neural-network-based parametrization in which case $\underline{\theta }$ can be seen as the set of weights and biases of the network^50,51^. We assume that possible choices for $\underline{\theta }$ lie in a set *Θ*, which is informed by the prior information we have about the data. The most common example of such a *Θ* is the *ℓ*_1_ ball, which is often chosen to enforce sparsity in learning tasks. Furthermore we demand that the true model lies inside this hypothesis set, i.e., that there is a ${\underline{\theta }}^{* }\in \Theta$ such that $E(\underline{\sigma },{\underline{\theta }}^{* })={E}^{* }(\underline{\sigma })$.

Given a parametric energy function, this defines a parametric distribution $p(\underline{\sigma },\underline{\theta })\propto \exp (E(\underline{\sigma },\underline{\theta })),$ on the $\underline{\sigma }$ variables. This in turn defines the following paramteric single-variable conditionals,

12 $$p({\sigma }_{u}=q| {\underline{\sigma }}_{\setminus u};\underline{\theta })=\frac{1}{1+{\sum }_{p\in [Q],p\ne q}{e}^{E({\underline{\sigma }}_{u\to p},\underline{\theta })-E({\underline{\sigma }}_{u\to q},\underline{\theta })}}$$

Now given *M* independent samples ${\underline{\sigma }}^{(1)},{\underline{\sigma }}^{(2)},\ldots {\underline{\sigma }}^{(M)}$ each drawn from a metastable distribution *ν*, the PL estimate for the model parameters is given by minimizing the average negative log-likelihood of the single-variable conditionals.

13 $$\underline{\hat{\theta }}={{\rm{argmin}}}_{\underline{\theta }\in \Theta }\frac{1}{M}{\sum }_{u=1}^{n}{\sum }_{t=1}^{M}\,{{{\mathcal{L}}}}_{u}(\underline{\theta },{\underline{\sigma }}^{(t)}).$$

14 $${{{{\mathcal{L}}}}}_{u}(\underline{\theta },\underline{\sigma }):=\log \left(1+{\sum}_{p\in [Q],p\ne {\sigma }_{u}}{e}^{E({\underline{\sigma }}_{u\to p},\underline{\theta })-E(\underline{\sigma },\underline{\theta })}\right).$$

Now, if the samples came from *μ*, it is well established that this is a consistent estimator of the model parameter^16,17,22^. Our results in the preceding section suggest that this is still a good estimator if the samples come from a strongly metastable state. We can rigorously show that this is indeed the case rather easily if we assume the following condition on the parametric energy function,

#### Condition 2

(Finite interaction strength) For every $\underline{\theta }\in \Theta$ (including ${\underline{\theta }}^{* }$), the change in energy function caused by perturbing the value of $\underline{\sigma }$ at a single site *u* ∈ [*n*] is upper bounded as follows,

15 $$\frac{1}{2}| E(\underline{\sigma },\underline{\theta })-E({\underline{\sigma }}_{u\to q},\underline{\theta })| \le \gamma,\,\,\forall \underline{\sigma }\in {Q}^{n},q\in Q,u\in [n].$$

For the case of polynomial energy functions often considered in the rigorous learning theory literature, this bound essentially boils down to an upper bound on the *ℓ*_1_ norm of the parametric coefficients multiplying the model. Further, for the specific case of Ising model energy functions defined on bounded degree graphs, the *γ* here will just be the product of an inverse temperature of the model and the degree of the graph.

The finite interaction strength condition directly leads to the following bound on the single-variable conditionals,

16 $$\frac{1}{1+(| Q| -1){e}^{2\gamma }}\le p({\sigma }_{u}| {\underline{\sigma }}_{\setminus u},\underline{\theta })\le \frac{1}{1+(| Q| -1){e}^{-2\gamma }}.$$

Given this bound on the conditionals and, in turn, on the conditional likelihood, we can bound the test error of the PL method. For the parameters $\underline{\hat{\theta }}$ learned from samples drawn from a metastable state, we can bound the test deviation of the PL loss function at $\underline{\hat{\theta }}$ from the true model at ${\underline{\theta }}^{* }.$ We state the formal theorem below, the proof of which can be found in Supplementary Note 6.

#### Theorem 2

(Test error for logistic regression with metastable samples) Given ${M}^{{\prime} }$ independent samples ${\underline{\sigma }}^{(1)},\ldots,{\underline{\sigma }}^{({M}^{{\prime} })}$, from an *η*-strongly metastable distribution *ν* of a reversible Markov chain, the true graphical model parameters are nearly optimal for PL in the following sense,

17 $$\begin{array}{l}\frac{1}{{M}^{{\prime} }}{\sum}_{t,u}{{{\mathcal{L}}}}_{u}({\underline{\theta }}^{ * },{\underline{\sigma }}^{(t)})-{{{\mathcal{L}}}}_{u}(\underline{\hat{\theta }},{\underline{\sigma }}^{(t)})\le \frac{2(1+(| Q| -1){e}^{2\gamma })\eta }{{\omega }_{P}}+\\ \log ({e}^{2\gamma }(| Q| -1))\sqrt{\frac{\log (\frac{1}{\delta })}{2{M}^{{\prime} }}}.\end{array}$$

Here the quantities *ω*_*P*_ and *γ* are related to the Markov chain and the prior in the PL estimation via conditions 1 and 2.

The $O(1/\sqrt{{M}^{{\prime} }})$ term here is the statistical error in the estimation of the loss function. Hence, if ${M}^{{\prime} }$ is small enough, this statistical error will swamp the *O*(*η*/*ω*_*P*_) bias introduced by having bad data. Looking at the connection between *η* and mixing times, we can see that for slow-mixing Markov chains, this bias can be exponentially small in *n*. Hence, we can conclude that there is a family of strongly metastable distributions for which the PL estimate of the energy function ($\underline{\hat{\theta }}$) asymptotically (*n*, *M* → *∞*) approaches the true model parameters (${\underline{\theta }}^{* }$). This observation is somewhat surprising, as this tells us that the true model can be nearly reconstructed from “bad” data using the PL estimator.

Theorem 2 is a very general result that applies to any discrete distribution as long as the two conditions are satisfied. However, this result by itself does not guarantee that the estimated parameters from Eq. 13 will closely match that of the true distribution. To give such learning guarantees, we have to show that the loss function has sufficiently large curvature near the optimal point. This would then imply that the small changes in the loss values translate to small changes in the estimated parameters. (See Fig. 2 of ref. ^52^ for a visual explanation of this point).

**Fig. 2:**
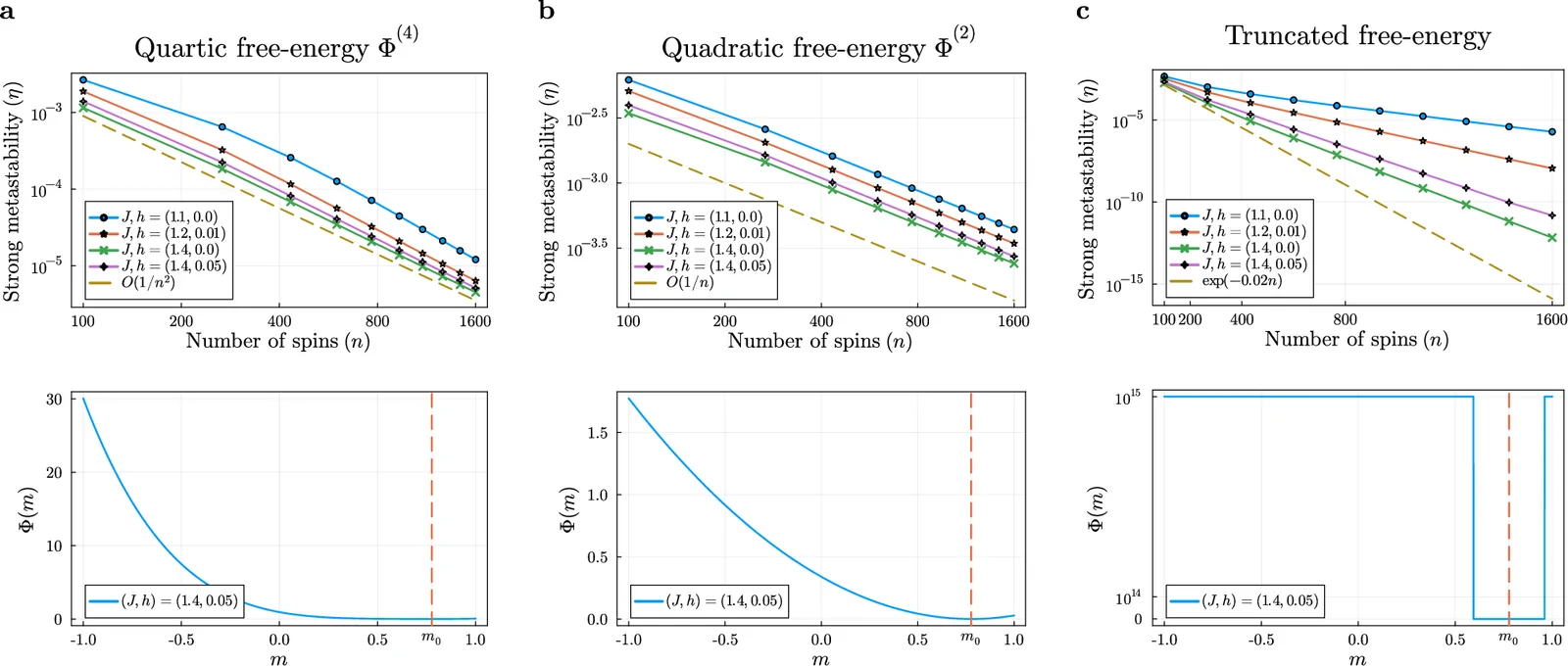
Strongly metastable states in the CW model. Here, we plot the violation in the detailed balance condition as defined in Eq. 3 computed by projecting to the magnetization space as in Eq. 78. **a** Fourth-order approximation to the free energy at *m*_0_. **b** Second-order approximation. **c** Truncated free energy as defined in Eq. 79.

### Parameter learning guarantees for Ising models

We can exploit the properties of single-variable conditionals of strongly metastable states to prove guarantees on parameter and structure recovery for models with up to pairwise interactions, i.e., Ising models with magnetic field terms. The true energy function then has the form,

18 $${E}^{* }(\underline{\sigma })={\sum}_{i < j}{\theta }_{ij}^{* }{\sigma }_{i}{\sigma }_{j}+{\sum}_{i=1}^{n}{\theta }_{i}^{* }{\sigma }_{i},\,\,{\sigma }_{i}\in \{-1,1\}$$

The learning task here is to estimate the parameters ${\underline{\theta }}^{* }$ from data. For this task, we will choose the parametric family to be the most general quadratic energy function on spin variables, $E(\underline{\sigma },\underline{\theta })={\sum}_{i < j}{\theta }_{ij}{\sigma }_{i}{\sigma }_{j}+{\sum }_{i=1}^{n}{\theta }_{i}{\sigma }_{i}.$ For these models, a sparse prior is imposed on the learning by imposing an *ℓ*_1_ constraint for each variable in the model^16^. Using this setup, a PL estimate for all the parameters connected to the variable at position *u* can be estimated as follows,

19 $${\underline{\hat{\theta }}}_{u}={{{\rm{argmin}}}}_{{| | {\underline{\theta }}_{u}| |}_{1}\le \gamma \,}\frac{1}{M}\mathop{\sum}_{t=1}^{M}\,\log (1+\exp (-2{\sigma }_{u}^{(t)}({\sum}_{j\ne u}{\theta }_{uj}{\sigma }_{j}^{(t)}+{\theta }_{u}))).$$

Here we use the shorthand ${\underline{\theta }}_{u}$ to denote all the coefficients connected to the variable at *u*, i.e., ${\underline{\theta }}_{u}=\{{\theta }_{u},{\theta }_{u1},{\theta }_{u2},\ldots,{\theta }_{un}\}.$ Notice that compared to Eq. 13, we have split the single optimization problem to *n* smaller optimizations. This is mainly done to make the ensuing theoretical analysis easier.

This type of estimator was first proposed by Besag^22^ as an efficient method for performing parametric estimation on high-dimensional data. The sample complexity of this method for the inverse Ising problem have been thoroughly investigated much more recently^16–18,31^ in the setting where i.i.d. samples are available from the true model.

Below, we state the learning guarantee on the error between the pairwise parameters learned from metastable samples and the true parameters of the energy function.

#### Theorem 3

(Learning pair-wise couplings) For $M=\lceil {2}^{10}\frac{{e}^{8\gamma }{\gamma }^{4}}{{\varepsilon }^{4}}{{\mathrm{log}}}(\frac{8n}{{\rm\delta }})\rceil$, samples drawn from an *η* strongly metastable distribution, with probability greater than 1 − δ the following guarantee holds for all *u* ∈ [*n*],

20 $${{\max }_{v\in [n],v\ne u}}| {\underline{\theta }}_{uv}^{* }-{\underline{\hat{\theta }}}_{uv}| \le \,\,\varepsilon+4{e}^{2\gamma }\sqrt{\frac{(1+\gamma )\eta }{{\omega }_{P}}}.$$

The proof of this result is provided in the Supplementary Note 7. This theorem can be seen as the metastable version of the learning guarantee for PL proved in ref. ^17^. Just like in the setting where we have samples from the true distribution, we see that the sample complexity is exponential in the *γ* parameter. But unlike these settings, there is an unavoidable bias in the error that does not go to zero as the number of samples increases. We can see from the constructions of strongly metastable states discussed before, this bias decays with the size of the system.

For Glauber dynamics, remember that *ω*_*P*_ = 1/*n*. So any metastable state with $\eta=o(\frac{1}{n})$ gives error guarantees with a bias that decays with system size.

An important problem associated with learning graphical models is *structure learning* ^53^, which refers to the reconstruction of the underlying graph structure of the model. To this end define the edge set *E* = {(*u*, *v*) ∈ [*n*] × [*n*]∣*θ*_*u*,*v*_ ≠ 0}. Then this edge set can be recovered from the learned couplings with high probability using a simple thresholding method.

#### Theorem 4

(Structure learning) Consider an Ising model with $| {\theta }_{uv}^{* }| > \alpha$ for every (*u*, *v*) ∈ *E*. Let $\underline{\hat{\theta }}$ be estimated using $M=\lceil {2}^{26}\frac{{e}^{8\gamma }{\gamma }^{4}}{{\alpha }^{4}}\log (\frac{8n}{\delta })\rceil$ samples from a *η*-strongly metastable state by solving the optimization problem in Eq. 19. Then if $\alpha > 16{e}^{2\gamma }\sqrt{\frac{(1+\gamma )\eta }{{\omega }_{P}}}$, the estimated edge set,

21 $$\hat{E}=\{(u,v)\in [n]\times [n]\,| \,\max (| {\hat{\theta }}_{uv}|,| {\hat{\theta }}_{vu}| ) > \alpha /2\}$$

will match *E* with probability greater than 1 − δ.

The assumed *η*-dependent lower bound on *α* is necessary to prevent the inherent bias in the learning from swamping very small nonzero couplings in the model. Even in cases where the bias is larger than the smallest coupling, this type of thresholding will correctly find the edges with larger weights.

Finally, we will show how the magnetic field can be provably recovered for *d*-sparse models. We achieve this by doing a second round of optimization after structure learning. This is done by estimating the second order terms first as in Theorem 3, reconstructing the structure and then optimizing the PL loss for a second round with only the magnetic field terms as the variable.

#### Theorem 5

(Recovering magnetic fields) Suppose that we are given the edge set *E* and estimates for all the pairwise parameters in the model such that $| | {\underline{\theta }}_{\setminus u}^{* }-{\underline{\hat{\theta }}}_{\setminus u}| {| }_{\infty }\le \varepsilon$. Further assume that the underlying graph has max degree of *d* and that $| {\theta }_{u}^{* }| \le {h}_{max}$. Then given samples from an *η*-strongly metastable state we can estimate the magnetic fields for each *u* as follows;

22 $${\hat{\theta }}_{u}={{{\rm{argmin}}}_{| {\theta }_{u}| \le {h}_{max}}}\,\frac{1}{M}{\sum}_{t=1}^{M}\,\log \left(\,1+\exp \left(-2{\sigma }_{u}^{(t)}\left({\sum}_{v:(u,v) \in E}\left\langle\underline{\hat{\theta }}_{u,v},{\underline{\sigma }}_{v}^{(t)} \,\right\rangle+{\theta }_{u}\right)\right).\right.$$

For $M=\lceil \frac{{2}^{7}{h}_{max}^{4}{e}^{4{h}_{max}}}{{\varepsilon }_{h}^{4}}\log (\frac{4}{\delta })\rceil$ we can guarantee with probability at least 1 − δ that the error in this estimate will be bounded as, $| {\hat{\theta }}_{u}-{\theta }_{u}^{* }| \le {\varepsilon }_{h}+4\sqrt{d\varepsilon {h}_{max}}{e}^{{h}_{max}}+4\sqrt{\frac{\eta {h}_{max}}{{\omega }_{P}}}{e}^{{h}_{max}}.$

Once again, we see that there is a bias term in the error guarantee that has its source in the metastability of the data distribution. The three theorems in this section together imply that every parameter in a *d*-sparse Ising model can be recovered with a small bias with sample complexity scaling as O(log(n)), generalizing the high-dimensional results from prior works to the case of metastable samples. Their complete proofs can be found in Supplementary Note 7.

### Strongly metastable distributions in the Curie–Weiss model

In this section, we illustrate some of the abstract results and constructions from the earlier sections to the specific case of the CW model. This model is defined by the following energy function on *n* binary spin variables (*σ*_*i*_ ∈ {1, −1}) parameterized by a coupling constant *J* and a magnetic field *h*,

23 $$E(\underline{\sigma })=\frac{J}{n}{\sum}_{i=1}^{n}{\sum}_{j=i+1}^{n}{\sigma }_{i}{\sigma }_{j}-h{\sum}_{i}{\sigma }_{i}.$$

This model is widely studied in statistical physics, where it serves as a canonical model for understanding symmetry-breaking phase transitions. The all-to-all connectivity of this model makes it an ideal candidate for theoretical and numerical explorations^4,54^. We can construct explicit examples of strongly metastable states with small *η* in the CW model. The CW model has permutation invariance, i.e., the probability of observing a certain configuration of spins in this model only depends on their total sum. Define the *magnetization* of a spin configuration as $m(\underline{\sigma })={\sum }_{i}{\sigma }_{i}/n$. Then the probability of observing a configuration with magnetization *m* is proportional to *e*^−*nΨ*(*m*)^, where the *free energy* *Ψ*(*m*) of the model

24 $$\Psi (m)=\frac{-J}{2}{m}^{2}+hm-S(m),$$

dictates the essential statistical properties of this system. Here $$S(m)=\frac{1}{n}\log \left(\begin{array}{l}n\\ \frac{n(1+m)}{2}\end{array}\right)$$ is the entropy term. The minima of this free energy function also correspond to regions in the state space from which Glauber dynamics struggles to escape^1^.

The approach taken here follows a classical paradigm from mean-field statistical mechanics and phase-transition theory: one studies the free energy as a function of the relevant order parameter and analyzes its local expansion near its minima. For the CW model, the magnetization is the natural order parameter, and the minima of *Ψ*(*m*) correspond to the thermodynamic phases of the model. A local expansion of *Ψ*(*m*) around these minima therefore describes the local concentration of the Gibbs measure, making it a natural starting point for the construction of metastable states. This is exactly the perspective underlying Landau-type descriptions of phase transitions and standard analyses of the CW free energy landscape^55,56^.

Inspired by the statistical physics picture, we look for candidate metastable states by analyzing *Ψ*(*m*). We motivate this method of construction of metastable states further in Supplementary Note 3 by matching the conditionals of candidate metastable states with that of the true distribution. There, we find that the metastable states that closely match the conditionals of the true distribution are centered around the minima of the free energy. Now, define *m*_0_ to be the positive value where the gradient of the free energy vanishes,

25 $$-J{m}_{0}+h-{S}^{{\prime} }({m}_{0})=0,\,\,{m}_{0}\ge 0.$$

We can construct candidate metastable states by using a *K*-order Taylor approximation of the free energy around this minimum point. Notice that the first-order terms vanish, and the constant term can be ignored as we are working with energies. These candidate metastable states can be defined by a free energy function fixed by the order of the expansion as follows,

26 $${\Phi }^{(K)}(m)\equiv {\sum}_{k=2}^{K}{\Psi }^{(k)}({m}_{0})\frac{{(m-{m}_{0})}^{k}}{k!}$$

Here, *Ψ*^(*k*)^ is the *k*-th derivative of the free energy. This metastable free energy defines a metastable distribution as follows,

27 $${\nu }^{(K)}(\underline{\sigma })\propto \frac{{e}^{-n{\Phi }^{(K)}(m(\underline{\sigma }))}}{\left(\begin{array}{l}n\\ \frac{n(1+m(\underline{\sigma }))}{2}\end{array}\right)}.$$

Notice that these metastable distributions are also permutation invariant. The violation of the detailed balance condition used in Eq. 3 is difficult to compute for general distributions. However, for permutation-invariant distributions, this quantity can be efficiently computed by first mapping the problem entirely onto the magnetization space. We give the details of this mapping in the Supplementary Note 3. Now, by numerically evaluating the detailed balance violation, we conclude that the *ν*^(4)^ distribution is $O(\frac{1}{{n}^{2}})$ strongly metastable for an *n-*spin Curie-Weiss model for *J* > 1 and 0 < *h* < 0.05. We also find that truncating the free energy exactly around *m*_0_ with a well-chosen width leads to *e*^−*O*(*n*)^—strongly metastable states, akin to the construction in Subsection “Existence of Strongly Metastable Distributions for Reversible Markov Chains”. The results of these experiments are given in Fig. 2.

### Numerical experiments on learning the Curie–Weiss model

In this section, we numerically confirm that the *J* and *h* parameters of the CW model can be learned given samples coming from a metastable state of a Markov chain.

We sample from this model using the Markov chain method known as Glauber dynamics^57^ and then attempt to learn the parameters *J* and *h* using the pseudo-likelihood method. The CW model is well-suited for this exploration for two reasons. Firstly, in the right parameter range, this model develops two metastable distributions^1,4^. By tuning the magnetic field *h,* we can also change the relative size (in terms of probability) of these two states. Secondly, because of the all-to-all, uniform couplings, we can run Glauber dynamics on very large system sizes.

**Fig. 3:**
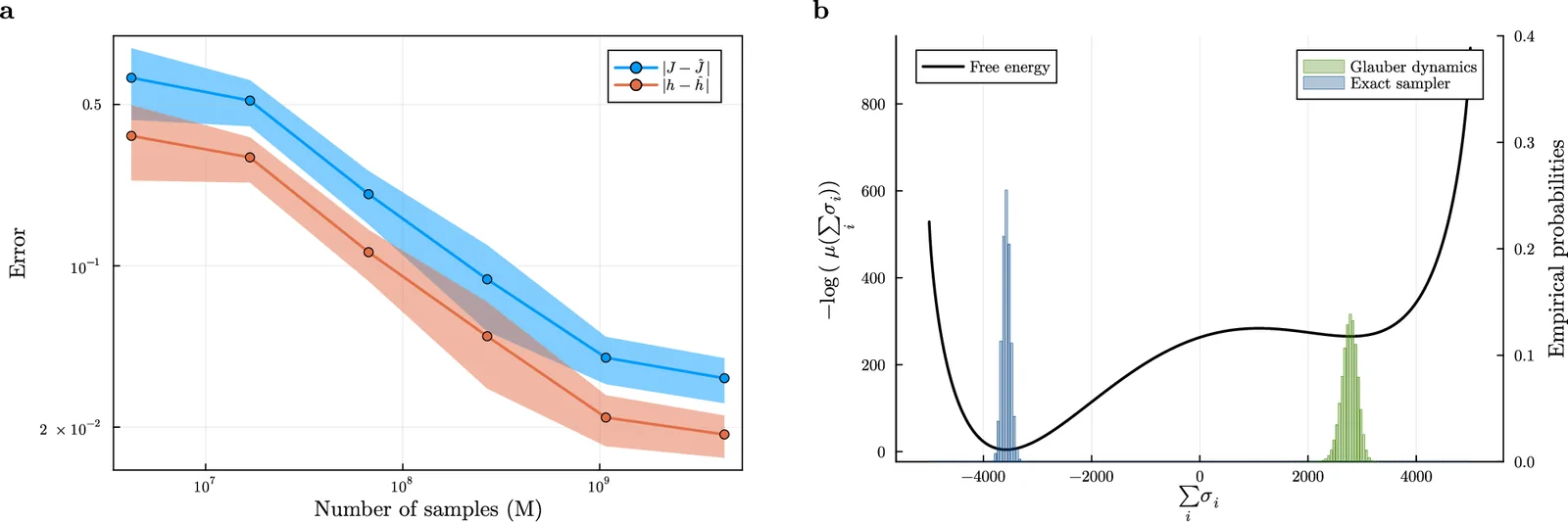
Learning the CW model. **a** Error in learning the Curie–Weiss model on 5000 spins. Samples here are produced by Glauber dynamics “stuck” at the positive minima of the free energy. True parameters here are *J* = 1.2, *h* = 0.04. **b** The true distribution is highly biased towards negative magnetization, as seen by the free energy curve. There is a metastable distribution with positive magnetization that is highly suppressed in terms of probability. The empirical distributions of samples (*M* = 4 × 10^9^) drawn by an exact sampler and Glauber dynamics is overlaid on top of the free energy. This shows that the Markov chain is effectively stuck around the positive minima.

In this experiment, we choose the system parameters such that the distribution is bimodal but heavily lopsided, with most of the probability in states that have negative magnetization. From Fig. 3b, we see that if i.i.d. samples were produced, the chance of observing a sample with positive magnetization (∑_*i*_*σ*_*i*_ > 0) will essentially be zero. But if we use Glauber dynamics to sample from this model and start the Markov chain from the all-one state (*σ*_*i*_ = 1 ∀ *i*), then the dynamics will get stuck in the positive magnetization part of the distribution. This is a well-studied property of CW models that can be rigorously established^4^. We corroborate this by plotting the empirical probabilities produced by an exact sampler and Glauber dynamics in Fig. 3b. This shows that Glauber dynamics is stuck around the minima with positive magnetization. At this point, we assume that the distribution of the samples generated by Glauber dynamics is close to a metastable distribution with positive magnetization. Notice that by any global metric (TV, KL divergence, etc.), this distribution is very far from the Gibbs distribution of the true CW model. This implies that a technique like maximum likelihood estimation (MLE) will not succeed in recovering the right model parameters ( *J* and *h*) as it empirically minimizes the KL-divergence between the samples and the parametric model we are trying to learn.

**Fig. 4:**
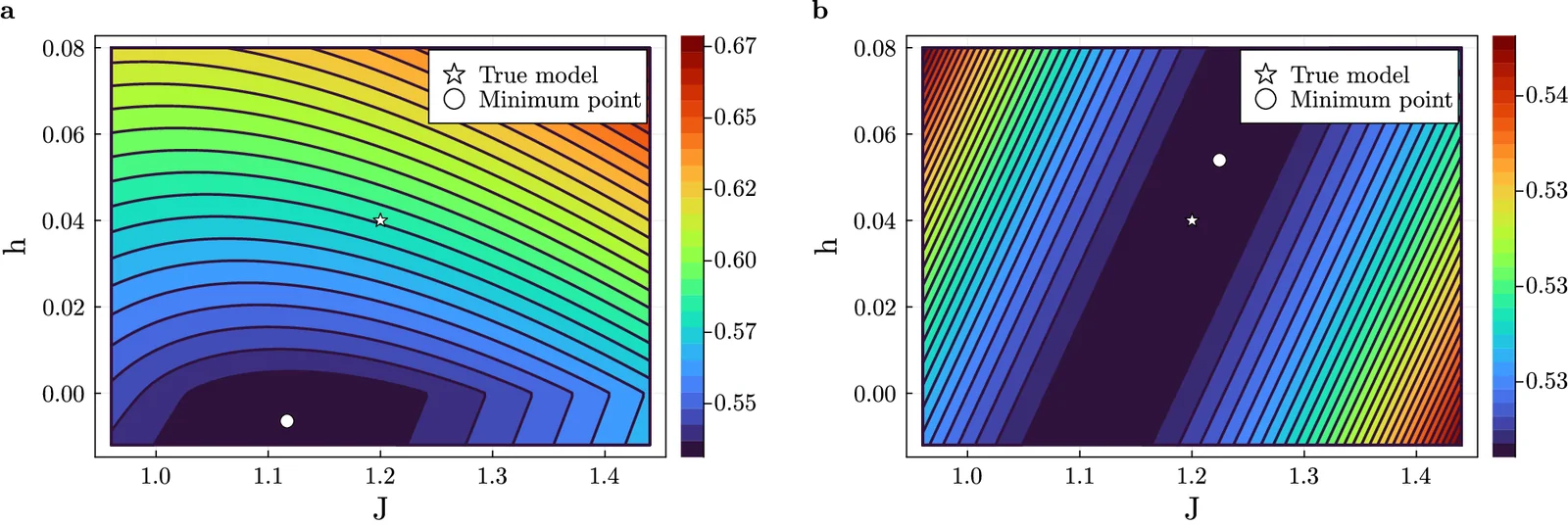
Comparison of the loss function landscape for the CW model with true parameters *J* = 1.2, *h* = 0.04. These are plotted with *M* = 2^32^ samples produced by Glauber dynamics “stuck” at the positive minima of the free energy. **a** Negative log-likelihood computed from this data clearly has its minimum far from the true model. The sign of the magnetization is the opposite of the true model. This is expected as maximum likelihood tries to match the sufficient statistics of the data to the model. **b** PL loss function has the minima close to the true model and learns the magnetic field with the right sign.

However, we find that the original model parameters can be recovered from these samples using the PL estimator. The results of learning using PL are shown in Fig. 3a, which shows that *J* and *h* can be learned from samples from the metastable distributions with remarkable accuracy. Given these samples, the correct values of *J*, *h* cannot be predicted by the maximum likelihood method. We demonstrate this in Fig. 4 by plotting the negative log-likelihood in the (*J*, *h*) space. We see that the minimum of this function lies far away from the true model. Figure 4 shows the PL loss function. We see from these plots that the PL loss function has its minimum close to the true model, while MLE predicts the wrong sign for the magnetic field in the model.

### Numerical experiments on learning a spin glass model

To demonstrate learning from metastable samples in the most general setting, we study this learning problem on a model with *q* > 2, higher-order interactions and glassy behavior. We choose our energy function as the sum of third-order terms over a hypergraph specified by the edge set E,

28 $$E(\underline{\sigma })=-\beta {\sum}_{(i,j,k)\in {\mathtt{E}}}\,W({\sigma }_{i},{\sigma }_{j},{\sigma }_{k}).$$

Here, each variable, *σ*_*i*_, can take values from {1,…,*q*}. The tensor *W* is a *q* × *q* × *q* tensor that determines how each hyperedge contributes to the total energy.

The tensor *W* is defined in the following way: let *a*, *b*, *c* ∈ {1,…,*q*} denote *q*-state variables. We define

*W*(*a*, *b*, *c*) ∈ {+1, −1} by

$$W(a,b,c)=\left\{\begin{array}{l}+1,\,{{{\rm{for}}}}\,(a,b,c)\in {\{(1,r,r),(r,1,r),(r,r,1)\}}_{r=1}^{q},\\ -1,\,{{{\rm{otherwise}}}}.\hfill\end{array}\right.$$

Equivalently, the only favored local configurations are those with one index equal to the distinguished state 1 and the remaining two indices equal.

To understand the behavior of this model, first let us look at the special case of *q* = 2. We can switch to the spin representation ({1, −1} alphabet) and equivalently write this energy function as, $E(\underline{\sigma })=-\beta {\sum }_{(i,j,k)\in {\mathtt{E}}}\,{\sigma }_{i}{\sigma }_{j}{\sigma }_{k}$. This shows that the model is not frustrated in the traditional sense; it has a ground state of all ones. However, this form of a three-body ferromagnetic interaction has been studied^58,59^, as an example of a system showing glassy behavior despite not being frustrated in the traditional sense. When this model is sampled using Glauber dynamics from a random initial state for high enough *β* on a hypergraph of sufficient edge density, the dynamics is seen to get stuck at a higher energy compared to the equilibrium distribution^59,60^. As pointed out in ref. ^58^, this is caused by the presence of a large number of metastable states that prevent the mixing of the dynamics to the stationary distribution of the Markov chain.

To demonstrate the effectiveness of learning from metastable distributions in the most general setting, we study the parameter and structure learning problem on these models at *q* = 3. For the underlying graph, we randomly choose a third-order hypergraph with a number of edges ∣*E*∣ = 3*n*/2.

**Fig. 5:**
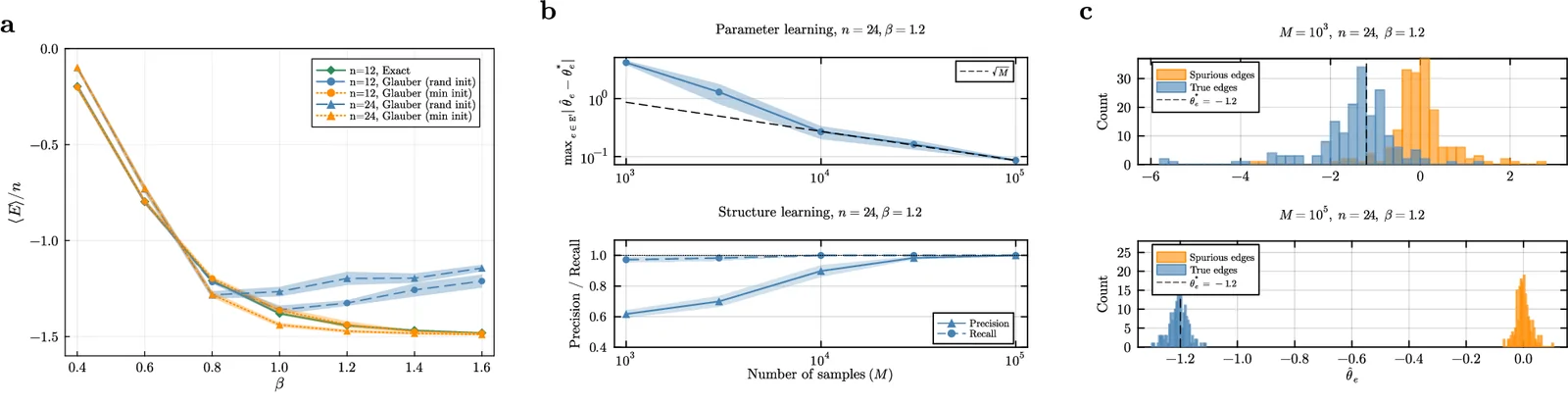
Learning from metastable samples for a spin glass model with *q* = 3. All datapoints are averaged over 5 different instantiations of the random hypergraph as specified in Eq. 28. **a** Metastability in this model can be observed by comparing the average energy of the samples produced by different samplers^58,60^. Glauber dynamics initialized from a random configuration is seen to get stuck at a higher energy than the exact sampler, and the same Glauber dynamics sampler started from the unfrustrated ground state of the model. For these experiments *M* = 10^5^. **b** Learning results from metastable samples collected from *n* = 24, *β* = 1.2 model. For structure learning, via thresholding, we choose to declare as edges all terms with a learned coupling greater than 0.12 in absolute value as the edges. The precision and recall of predicting a hyperedge using this threshold is reported in the bottom panel (**c**). Shows the histogram of learned couplings for different values of *M*. All chains in these experiments are restarted 16 times during the sampling, with only the tenth sample kept till the desired number of samples are collected. Each restart is initialized with a burn-in of 10^4^ steps.

Similar to the *q* = 2 case studied in literature, we observe that for the *q* = 3 model, as *β* increases, Glauber dynamics starting from a random initial state is seen to get stuck at a higher average energy than that is given by the exact sampler. In Fig. 5a, we study the average energy as a function of *β* and observe this onset of metastability. Since the all-one state is a ground state, starting Glauber dynamics from this state is seen to match the results of the exact sampler at *n* = 12. Since exact sampling at *n* = 24 is not viable, we use the fact that the randomly initialized Glauber being stuck at a higher energy than the minimum initialized Glauber as an indication of metastability. Now, to test our learning algorithm, we use the samples generated from the *n* = 24 Glauber chain with random initialization as samples. The chain is restarted 16 times for this experiment, and only every tenth sample is recorded from the chain to ensure that the dynamical effects of the sampler are reduced in the dataset fed to the learning algorithm. In the PLE loss we use a hypothesis energy function of the form,

29 $$E(\underline{\sigma },\underline{\theta })=-{\sum}_{(i,j,k)\in {{\mathtt{E}}}^{{\prime} }}\,{\theta }_{i,j,k}\,W({\sigma }_{i},{\sigma }_{j},{\sigma }_{k}),$$

such that this hypergraph has twice the number of edges as the true model and is its strict superset, i.e., $| {{\mathtt{E}}}^{{\prime} }|=3n$ and ${\mathtt{E}}\subset {{\mathtt{E}}}^{{\prime} }$. We do not use the prior information that all the edges have uniform couplings; instead, the learning is done with an *ℓ*_1_ constraint that will not enforce any uniformity. The results of the parameter and structure learning experiments are given in Fig. 5b, averaged over 5 different random instantiations of the hypergraph underlying the model. These show that both these tasks succeed given metastable samples. Figure 5c shows the histogram of the couplings learned from these metastable states. This shows that with enough samples, the learning algorithm is clearly able to classify the hyperedges present in the energy function from the non-edges.

## Discussion

Metastability of stochastic dynamics is an important idea that lies at the heart of many interesting phenomena studied in statistical physics. In this work, we have shown that metastability has important effects on learning from discrete data. Our work opens up several interesting directions for exploration. For instance, it is natural to ask if there are large separations in the two measures of metastability defined in this work. In Supplementary Note 2, we show that for a given *P*, the largest separation between these measures is at least as big as the diameter of the underlying graph of transitions. We conjecture that this is not the optimal separation between these two measures of metastability. If this separation is indeed tight, then that implies only an *O*(*n*) difference between the two measures of measures for the Glauber dynamics Markov chain. This is because the underlying graph of transitions for Glauber dynamics is the *n*-bit hypercube, which has a diameter of *n*. This would then imply non-trivial learning guarantees for all metastable states. The connections between these notions and electrical networks used in the proof of Proposition 4 might be useful for these explorations. The closeness of conditionals implied by strong metastability in Theorem 1 is akin to the Dobrushin–Lanford–Ruelle (DLR) condition used in the definition of infinite-volume Gibbs measures^61^. However, the family of distributions defined by a strongly metastability condition, with an appropriately vanishing *η* with system size, is richer than what is defined by the DLR condition.

There is also a natural question of whether strong metastability, as defined in this paper, is the right model for describing samples collected from an unmixed Markov chain. A more refined question is whether one can guarantee, under suitable conditions, that the Markov chain samples from a strongly metastable state when run for a length of time much smaller that the mixing time. These are relevant questions not only for Gibbs distribution learning, but also for analyzing the performance of a broad class of algorithms, and hence has been a subject of interest in recent literature. For instance, Liu et al.^37^, problem 1.23 posed this question as an open problem for a natural class of strongly metastable states. The recent work of Alman et al.^62^, Theorem 5 rigorously formalizes a conditional version of this phenomenon for random walks on graphs: the walk can mix locally, after conditioning on a nonnegligible-probability event, in regimes where the unconditioned walk need not yet be globally mixed. Extending these explorations to general reversible Markov chains would be directly relevant to the results presented here.

Another point concerns how samples are collected in a typical setting. A discerning reader might argue that an i.i.d. sample from a metastable state is never produced while evolving the chain in a strict sense, since the distribution continues to evolve toward equilibrium. There are two responses to this concern. First, when the chain is trapped in a low-conductance set, this evolution is very slow, and hence the samples collected from the evolving chain are well modeled by independent samples from a metastable distribution. Second, if the sampler can be reset, as is possible in many settings, one can reset the state, run the chain for the same amount of time, and obtain an independent sample from the same distribution. Moreover, as shown by the results in Section, “Existence of Strongly Metastable Distributions for Reversible Markov Chains,” strong metastability is fairly robust to errors in time of collection and mixing with samples from well-separated modes. This robustness is also reflected in our experiments, where we collect a large number of samples while restarting the chain only 16 times. This setting is more natural than the one considered in previous Markov-chain learning papers, since we do not require samples to be collected at every time step^29^.

The learning guarantees for the Ising case presented here can be generalized to more general models, as done in prior works on learning graphical models^20,21^. Notice that while we have focused on the pseudo-likelihood method, the same arguments presented here would be applicable to any method that exploits conditionals for learning, like interaction screening^19^ or Sparsitron^21^. In fact, we believe that similar ideas will even carry over to the case of continuous alphabets.

Improvements on our sample complexity results may be possible focusing on a specific class of models, like Sherrington–Kirkpatrick models^63^ or low-rank Ising models^64^. Following these works that deal with i.i.d samples from the stationary distribution, for these specific families, exponential dependence on the *ℓ*_1_ width for metastable learning may be improved. However, in general, this dependence is unavoidable from the information-theoretic lower bound^49^. Extension of our results to continuous variable graphical models is also an interesting direction to consider. However, the state of sample complexity results in this setting is not as mature as that for the discrete setting, even when the samples come from the stationary distribution^65^.

Viewing the results of this work from a higher level, we have established that generalization is possible in the context of learning discrete distributions even if the data is constrained to come from some specific regions of state space. This generalization is made possible by the use of methods that learn the conditionals and by priors imposed during learning (in terms of the low-degree form of the energy assumed during learning and the constraints used in the optimization). This demonstrates an interplay between algorithms and prior, which allows learning to succeed even in the presence of the “wrong” data. The conditional-based methods used here are not limited to graphical models; they can also be used, for instance, to learn energy-based models with a neural net parametrization of the energy^51^. The exploration of the ideas presented here in the context of more general energy-based models is expected to shed light on the observed generalization properties of such models.

## Methods

### Proof framework

All theoretical results are proved for finite-state reversible Markov chains. Proposition 1, the lower bound on mixing time from ordinary metastability, is proved using only the contraction of the TV distance under a Markov transition. In particular, for any *v*, ∥*vP*∥_1_ ≤ ∥*v*∥_1_. Applying this contraction repeatedly to the increments *νP*^*t *+ 1^ − *νP*^*t*^ shows that a chain started from an *η*-metastable distribution cannot move more than *η* in TV per step.

The construction of strongly metastable states from bottlenecks uses reversibility. Given a set $A\subset {{{\mathcal{S}}}}$, the stationary measure restricted to *A* has no internal detailed-balance defect: all currents between pairs of states inside *A* cancel exactly by reversibility of *μ*. The only remaining terms are currents crossing from *A* to *A*^*c*^. Hence, the strong metastability parameter of the restricted measure is the normalized probability flow across the boundary of *A*, i.e., the conductance of *A*. The Cheeger inequality for reversible chains is then used only as an existence result: if a reversible chain has a small spectral gap, it has a low-conductance set and therefore supports a strongly metastable distribution^42,46^. The separation between ordinary and strong metastability is proved by mapping a reversible chain to an electrical network^66,67^. The edge conductance is

$${c}_{xy}=\mu (x)P(\,y| x)=\mu (\,y)P(x| \,y),$$

and a distribution *ν* defines a voltage

$${V}_{\nu }(x)=\frac{\nu (x)}{\mu (x)}.$$

The associated electrical current is

$${I}_{\nu }(x,y)={c}_{xy}\left({V}_{\nu }(x)-{V}_{\nu }(y)\right).$$

Under this correspondence, ordinary metastability controls the net current injected or removed at each vertex, while strong metastability controls the total absolute current through the network. Choosing a source and sink at maximal graph distance forces the same net current to cross every graph sphere separating them. Summing over these spheres gives the diameter factor in Proposition 4. The required source–sink distribution is obtained by solving the corresponding Poisson equation and then adding a sufficiently large multiple of the stationary distribution to make the solution non-negative.

The main conditional-closeness theorem is proved by applying the strong metastability condition to only to one-site transitions. Reversibility gives

$$\frac{P({\underline{\sigma }}_{u\to a}| \underline{\sigma })}{P(\underline{\sigma }| {\underline{\sigma }}_{u\to a})}=\frac{\mu (a| {\underline{\sigma }}_{\setminus u})}{\mu ({\sigma }_{u}| {\underline{\sigma }}_{\setminus u})}.$$

The bounded spin-flip condition then converts the detailed-balance defect into a weighted discrepancy between the one-site conditional distributions of *ν* and *μ*. The remaining step is an elementary probability-vector inequality: for probability vectors *p*, *q*, and any coordinate *k*,

$$| {p}_{k}-{q}_{k}| \le {\sum}_{\ell \ne k}| {p}_{k}{q}_{\ell }-{q}_{k}{p}_{\ell }|.$$

Applying this inequality at every site yields Theorem 1. The KL-divergence version used for pseudo-likelihood follows from the reverse Pinsker inequality together with the finite-interaction-strength lower bound on all one-site probabilities^48^.

### Learning guarantees

The learning proofs are written as empirical-risk arguments for the pseudo-likelihood loss. For a fixed site *u*, let ${{{\mathcal{L}}}}({\underline{\theta }}_{u})$ be the population pseudo-likelihood risk under the metastable sampling distribution *ν*, and let ${{{{\mathcal{L}}}}}_{M}({\underline{\theta }}_{u})$ be its empirical version from *M* samples. The estimator is the constrained minimizer of ${{{{\mathcal{L}}}}}_{M}$ over the prescribed parameter set.

The first step is to show that the true parameter is nearly stationary for the population loss, even though the data are not drawn from the stationary distribution. In the Ising case, the gradient at the true parameter can be written as an average of the difference between the one-site conditionals of *ν* and *μ*. Theorem 1, therefore, bounds the population gradient by a term proportional to *η*/*ω*_*P*_. Hoeffding’s inequality gives the corresponding empirical gradient bound^68^.

The second step is a curvature lower bound. The sitewise Ising loss is the logistic loss

$$f(x)=\log (1+\exp (-2x)).$$

On the finite-temperature domain imposed by the *ℓ*_1_ constraint, this loss has uniformly positive curvature. The curvature of the pseudo-likelihood risk is then related to conditional variances under *ν*. These variances are compared to the corresponding variances under *μ* using the same conditional-closeness theorem.

Combining the bounds on the gradient and curvature leads to the following argument that is used in the *M*-estimator literature: the estimator cannot move far from the true parameter without increasing the empirical loss more than allowed by the upper bound on the gradient norm. A union bound over sites yields the stated high-probability parameter-recovery guarantee. The structure-learning theorem is then obtained by thresholding the recovered pairwise couplings. The magnetic-field guarantee uses the same argument after the pairwise structure has been fixed, so that only the one-dimensional field parameter at each site is optimized.

### Glauber dynamics

The numerical experiments use heat-bath Glauber dynamics ^57^. For a target distribution *μ* on *Q*^*n*^, one update chooses a site *u* uniformly from {1,…,*n*} and resamples its value from the exact conditional distribution

$$P({\underline{\sigma }}_{u\to a}| \underline{\sigma })=\frac{1}{n}\mu (a| {\underline{\sigma }}_{\setminus u})$$

for one-site moves. This sampler satisfies the bounded spin-flip condition with *ω*_*P*_ = 1/*n*.

The theoretical learning results assume independent samples from a fixed metastable distribution. The numerical experiments use long Markov-chain trajectories as an empirical test of this prediction. Burn-in, thinning and independent restarts are used to reduce serial dependence before the retained samples are passed to the learning algorithm.

### Evaluation metrics

Parameter error is reported as the maximum absolute error over the relevant learned interaction coefficients,

$$\parallel \underline{\hat{\theta }}-{\underline{\theta }}^{* }{\parallel }_{\infty }={{\max }_{e}}| {\hat{\theta }}_{e}-{\theta }_{e}^{* }|.$$

For candidate interactions that are not present in the true model, the true coefficient is taken to be zero.

Structure recovery is evaluated by thresholding the learned coefficients. After thresholding, true positives are candidate interactions that are both selected and present in the true model; false positives are selected candidate interactions absent from the true model; and false negatives are true interactions not selected by the threshold. Precision and recall are computed as

$${{{\rm{Precision}}}}=\frac{{{{\rm{TP}}}}}{{{{\rm{TP}}}}+{{{\rm{FP}}}}},\qquad {{{\rm{Recall}}}}=\frac{{{{\rm{TP}}}}}{{{{\rm{TP}}}}+{{{\rm{FN}}}}}.$$

The reported spin-glass curves are averages over independently generated random hypergraphs, with the same planted hypergraph used across the sample-size sweep within each trial.

### Numerical implementation

All numerical routines are implemented in Julia. The optimization problems for the pseudo-likelihood estimators are solved through using JuMP^69^ and Ipopt^70^ libraries. For the CW model, the code uses the one-dimensional magnetization representation for exact sampling, exact likelihood evaluation and pseudo-likelihood fitting. For the spin-glass model, the multi-alphabet pseudolikelihood objective is constructed from a histogram representation of the samples and then optimized under *ℓ*_1_ constraints.

## Supplementary information

Supplementary Information

Transparent Peer Review File

## Data Availability

The code used to generate the data used for numerical experiments can be found in this repository https://github.com/abhijithjlanl/metastable-learning.

## Code availability

Codes used for learning experiments can also be found here https://github.com/abhijithjlanl/metastable-learning. The documentation in this repository contains detailed information on how to recreate the experiments.
