# Supplementary material for: Discrete distributions are learnable from metastable samples
Source: Nat Commun. 2026 Jul 23;17:8957. doi: 10.1038/s41467-026-75439-1 (PMC13503862; doi:10.1038/s41467-026-75439-1)
Supplement: Supplementary file 1 [file 41467_2026_75439_MOESM1_ESM.pdf]

Supplementary Information

Discrete distributions are learnable from metastable samples

SUPPLEMENTARY NOTE 1: PROOF OF PROPOSITION 1

For any Markov chain P and any vector v the following data-processing inequality holds,

$$||vP||_1 = \sum_i |\sum_j P(i|j)v(j)| \leq \sum_i \sum_j P(i|j)|v(j)| = ||v||_1 \tag{31}$$

Now give an η metastable distribution ν, using the above data-processing inequality repeatedly on $v = \nu P - \nu$ gives us,

$$|\nu P^{k+1} - \nu P^k| \leq \eta \tag{32}$$

Now suppose that after T steps starting from ν the Markov chain gets ϵ close in TV to the equilibrium distribution μ. From this,

$$|\nu - \mu|_{TV} \leq |\nu - \nu P|_{TV} + |\nu P - \mu|_{TV}, \tag{33}$$

$$\leq |\nu - \nu P|_{TV} + |\nu P^2 - \nu P|_{TV} \ldots |\nu P^T - \nu P^{T-1}|_{TV} + |\nu P^T - \mu|_{TV}, \tag{34}$$

$$\leq \eta T + |\nu P^T - \mu|_{TV}. \tag{35}$$

This gives $|\nu P^T - \mu|_{TV} \geq |\nu - \mu|_{TV} - \eta T$.

Now $|\nu P^T - \mu|_{TV} \leq \epsilon$, gives us $T \geq \frac{|\nu-\mu|_{TV}-\epsilon}{\eta}$.

SUPPLEMENTARY NOTE 2: SEPARATIONS BETWEEN METASTABILITY AND STRONG METASTABILITY

The two definitions of metastability raises an interesting question: *For a reversible Markov chain does $\eta-$ metastability imply $\eta-$ strong metastability?* We can construct a simple counter-example showing that is not true. For an even integer L, let $\mathcal{S} = [L]$ and P be the Markov chain corresponding to the random walk on a cycle graph on L elements. Take ν to be the following 'tent' distribution,

$$\nu(i) = \frac{1}{Z}\begin{cases} iL, & i \leq L/2, \\ -iL + L^2, & i > L/2, \end{cases} \tag{36}$$

The Z here ensures normalization. Now by direct computation, we can verify that this state is $\frac{2L}{Z}$ metastable while being $\frac{L^2}{2Z}$ strongly metastable. This shows that there can be $\Theta(|\mathcal{S}|)$ factor difference between the two measures metastability. This construction can be generalized to general reversible Markov chains using by mapping them to an electrical network and analyzing the current flows.

Proposition 4. Let $G = (\mathcal{S}, E)$ be a graph defined on the state space with the edge set $E = \{(i,j)|P(i|j) \neq 0\}$. Let $diam(G)$ be the diameter of this graph. Then the chain P has a η- metastable distribution that is $\Omega(diam(G)\eta)-$strongly metastable.

We conjecture that this construction does not give the optimal separation between metastable distributions and strong metastable distributions. The reason is that the diameter is a topological property of the chain and it can be changed drastically by adding a few well-chosen very small but non-zero transitions to the chain.

While the relationship between these two notions of metastability is an interesting question, it is not the main focus of this paper. We leave further exploration of this for future work.

1. *Proof of Proposition 4*

Mapping the problem to an electric network: To prove this statement we first map the Markov chain to an electric network using the well-known mapping between these two problems[1, 2]. The conductance [2] between two state i, j will be given by,

$$c_{ij} = \mu(i)P(j|i) = \mu(j)P(i|j). \tag{37}$$

We also associate a voltage with each node,

$$V(i) = \frac{\nu(i)}{\mu(i)}. \tag{38}$$

Let E be the edges with non-zero conductance, $E = \{(i,j) \in \mathcal{S} \times \mathcal{S} |\ c_{ij} \neq 0\}$.

We also associate a current with every edge according to Ohm's law,

$$I(i,j) = (V(i) - V(j))c_{ij} \quad \forall (i,j) \in E \tag{39}$$

Note that this current is antisymmetric, that is, $I(i,j) = -I(i,j)$.

Now for two nodes s and t in the graph, pick an $\eta-$ metastable distribution such that $\nu(I-P) = \frac{\eta}{2}(\vec{e}_s - \vec{e}_t)$. In the last part of the proof we will show that such a state always exists for some $\eta > 0$. We leave the choice of s and t free for now, but later we will fix it to get optimal bounds. We can show that this metastable distribution maps to an electrical network that has current $\eta/2$ injected at s and extracted at t. Consider the following relations,

$$\nu(i) - \sum_j \nu(j)P(i|j) = \sum_j P(j|i)\nu(i) - \nu(j)P(i|j) = \sum_j c_{ij}(V(i) - V(j)) = \sum_{j:(i,j)\in E} I(i,j) \tag{40}$$

The above expression is just the total current flowing into i. From the metastability condition on ν we see that total current flow is conserved at all nodes except s and t. At these nodes current of $\eta/2$ is injected/extracted.

Now the strong metastability of the state can be shown to be measured by the total absolute value current flowing in the system,

$$\sum_{i,j} |P(j|i)\nu(i) - \nu(j)P(i|j)| = \sum_{i,j} c_{ij}|V(i) - V(j)| = \sum_{i,j\in E} |I(i,j)| \tag{41}$$

To bound this quantity we consider spheres of increasing size centred around the source and consider the current flow through their boundaries. We define these spheres and their boundaries using the graph distance $d_E(i,j)$ which we take to be the shortest path length that connects node i and j in the undirected graph $G(\mathcal{S}, E)$.

$$\partial B(s,r) = \{(i,j) \in E \mid d_E(s,i) = r,\ d_E(s,j) = r+1\}, \qquad \textit{(Boundary sets)} \tag{42}$$

$$B(s,r) = \{j \in \mathcal{S} \mid d_E(s,j) = r\}. \qquad \textit{(Sphere sets)} \tag{43}$$

---

[2] Not to be mistaken for the conductance of the Markov chain that is defined in context of probability flows. This conductance is the inverse of the electrical Resistance.

Notice that the boundary sets are directed. They do not contain both (i,j) and (j,i) edges.

s and t nodes are unspecified as of now. We take these to be the maximally separated nodes in the graph $G(\mathcal{S}, E)$, that is $d_E(s,t) = diam(G)$ Using this observation in (41),

$$\sum_{i,j} |P(j|i)\nu(i) - \nu(j)P(i|j)| \geq \sum_{r=0}^{diam(G)-1} \sum_{(i,j)\in\partial B(s,r)} |I(i,j)| \geq \sum_{r=0}^{diam(G)-1} \left| \sum_{(i,j)\in\partial B(s,r)} I(i,j) \right| \tag{44}$$

Now intuitively we expect that the total current flow through every boundary set must be the same due to current conservation law in (40). We show this rigorously below. From the conservation law, for any $0 < r < diam(G)$ (avoiding the source and sink)

$$0 = \sum_{i\in B(s,r)} \sum_{j:(i,j)\in E} I(i,j) \tag{45}$$

In the above equation the edges connecting nodes inside $B(s,r)$ will not contribute as both $I(i,j)$ and $I(j,i)$ will exist in the sum which cancel due to antisymmetry. The remaining edges either lie in $\partial B(s,r)$ or $\partial B(s,r-1)$. Hence,

$$0 = \sum_{i,j\in\partial B(s,r)} I(i,j) + \sum_{(j,i)\in\partial B(s,r-1)} I(i,j) \tag{46}$$

Now the total flow out of the source $\sum_{(i,j)\in\partial B(s,0)} I(i,j) = \sum_{(s,j)\in E} I(s,j) = \eta/2$. Using this relation iteratively in (46) we get that the current flow through every boundary is the same,

$$\left| \sum_{(i,j)\in\partial B(s,0)} I(i,j) \right| = \left| \sum_{(i,j)\in\partial B(s,1)} I(i,j) \right| = \ldots = \left| \sum_{(i,j)\in\partial B(s,diam(G)-1)} I(i,j) \right| = \eta/2 \tag{47}$$

Using these relation in (44), we get,

$$\sum_{i,j} |P(j|i)\nu(i) - \nu(j)P(i|j)| \geq \sum_{r=0}^{diam(G)-1} \sum_{(i,j)\in\partial B(s,r)} |I(i,j)| \geq diam(G)\eta/2 \tag{48}$$

Existence of s-t $\eta-$ metastable distribution The question remains whether there exists a distribution such that, $\nu(I-P) = \frac{\eta}{2}(\vec{e}_s - \vec{e}_t)$. We will now show that such a distribution must always exist with some non-zero η.
First, observe that if P is an irreducible chain then the equilibrium distribution μ is the unique zero left-eigenvector of $I-P$. Moreover, in this case the by Perron–Frobenius theorem (refer Chapter 8, [3]) μ is a strictly positive vector.

For some $\eta' > 0$, consider the system of equations $\nu'(I-P) = \frac{\eta'}{2}(\vec{e}_s - \vec{e}_t)$. The matrix $I-P$ is not full rank, but its null space is only one dimensional as irreducible chain has a unique stationary state. The only zero right eigenvector of $I-P$ is the vector of all ones. $\frac{\eta'}{2}(\vec{e}_s - \vec{e}_t)$, is orthogonal to this vector. Hence this system will have a solution.

For any ν' satisfying this system and for any real α, the vector $\nu' + \alpha * \mu$ any is also a solution to the above linear system. Now we can take α to be a large enough value such that we hit a positive solution. Thus for any η', there always exists a positive vector $\nu' > 0$ such that $\nu'(I-P) = \frac{\eta'}{2}(\vec{e}_s - \vec{e}_t)$. Now given such an η' and ν' we can define,

$$\nu(i) = \nu'(i) / \sum_i \nu'(i) \tag{49}$$

$$\eta = \eta' / \sum_i \nu'(i) \tag{50}$$

We see that ν is a probability distribution which will be an $\eta-$metastable distribution, satisfying $\nu(I-P) = \frac{\eta}{2}(\vec{e}_s - \vec{e}_t)$. Since $\eta' > 0$ and ν' is positive, we have $\eta > 0$.

SUPPLEMENTARY NOTE 3: METASTABLE DISTRIBUTIONS OF CURIE-WEISS MODEL

Consider the order parameter magnetization per spin, $m(\underline{\sigma}) = \frac{\sum_i \sigma_i}{n}$.

The Gibbs distribution associated with the Curie-Weiss model can be expressed as,

$$\mu(\underline{\sigma}) = \sum_{m \in \{-1,-1+2/n,\ldots,1\}} \mu(\underline{\sigma}|m) \frac{\exp(-n\Psi(m))}{Z_\Psi} \tag{51}$$

Where $\mu(\underline{\sigma}|m)$ is the probability of observing the configuration $\underline{\sigma}$ given a fixed magnetization. For CW-type models with permutation symmetry, the probability of observing a certain configuration only depends on the magnetization and hence this is purely an entropic quantity, $\mu(\underline{\sigma}|m) = \delta_{m(\underline{\sigma}),m} \frac{1}{\binom{n}{\frac{n(1+m)}{2}}}$. The free energy function, $\Psi(m)$ fixes the distribution over the order parameters values. By direct inspection of the CW model Hamiltonian we can see that,

$$\Psi(m) = -\frac{J}{2}m^2 + hm - S(m). \tag{52}$$

Here $S(m) = \frac{1}{n}\log\binom{n}{\frac{n(1+m)}{2}}$ is the entropy term. This free energy is a well-studied quantity in statistical physics and defines the canonical model for the study of phase transitions [4]. It is also well-established that the Glauber dynamics for this model gets stuck at metastable distributions which are defined by minima of this free energy [5]. Now we will show that the minima of the free energy also corresponds to distributions that have the same single-variable conditionals as μ.

To this end, let us explore the following question; *Which other permutation invariant distributions will have the same single-variable conditional as* μ*?*

The relevance of this question comes from the fact that learning algorithms like PL learn the single-variable conditionals, the existence of such a distribution ν that matches μ approximately in single-variable conditionals implies that μ can be learned even if the parameters come from ν.

From the permutation invariance, we define ν using the following general form,

$$\nu(\underline{\sigma}) = \sum_m \mu(\underline{\sigma}|m) \frac{\exp(-n\Phi(m))}{Z_\Phi} \tag{53}$$

Now use the following relations, $\mu(\underline{\sigma}|m) = \frac{\mu(\underline{\sigma},m)}{\mu(m)} = \mu(\underline{\sigma})\delta_{m(\underline{\sigma}),m}\exp(n\Psi(m))Z_\Psi$,

$$\nu(\underline{\sigma}) = \mu(\underline{\sigma}) \frac{Z_\Psi}{Z_\Phi} e^{-n(\Phi(m)-\Psi(m))} \quad \delta_{m(\underline{\sigma}),m}. \tag{54}$$

Now remember that $\underline{\sigma}_{\neg i}$ is just $\underline{\sigma}$ with the $i-$th spin flipped. The single-variable conditionals of any distribution is simply $\nu(\sigma_i|\underline{\sigma}_{\setminus i}) = \frac{1}{1+\frac{\nu(\underline{\sigma}_{\neg i})}{\nu(\underline{\sigma})}}$. Hence these conditionals are fixed completely by the ratio of probabilities in the denominator. Let us now compute this ratio for ν. Without loss of generality we assume that $\sigma_i = 1$,

$$\frac{\nu(\underline{\sigma})}{\nu(\underline{\sigma}_{\neg i})} = \frac{\mu(\underline{\sigma})}{\mu(\underline{\sigma}_{\neg i})} e^{-n(\Phi(m)-\Phi(m-2/n)-(\Psi(m)-\Psi(m-2/n))} \quad \delta_{m(\underline{\sigma}),m}. \tag{55}$$

Now for the conditionals of these two distributions to approximately match the term in the exponent has to be close to zero. Enforcing this condition will give us the form of Φ which is the only unknown in the RHS.

To make the calculations easier, define the following finite difference operator of a function,

$$\Delta_n f(m) = f(m) - f(m - 2/n) \tag{56}$$

Then,

$$\Delta_n m = \frac{2}{n}, \tag{57}$$

$$\Delta_n m^2 = \frac{4m}{n} - \frac{4}{n^2}, \tag{58}$$

$$\Delta_n m^k = O(\frac{m^{k-1}}{n}). \tag{59}$$

Now we make the assume the following quadratic ansatz for $\Phi(m)$, for some $a > 0$ and $m_0 \in [-1, 1]$,

$$\Phi(m) = \frac{-a(m - m_0)^2}{2}$$

We have to determine a and m_0 such that the single-variable conditionals match as much as possible.

$$\Delta_n \Phi(m) = \frac{-2a(m - m_0)}{n} + \frac{2a}{n^2}. \tag{60}$$

Now for Ψ, we expand the free energy up to second order around the (as of yet unknown) point m_0,

$$\Psi(m) = -\frac{J}{2}m^2 + hm - S(m_0) - (m - m_0)S'(m_0) - \frac{(m - m_0)^2}{2}S''(m_0) + O((m - m_0)^3) \tag{61}$$

This gives,

$$\Delta_n \Psi(m) \approx -J(\frac{2m}{n} - \frac{2}{n^2}) + h\frac{2}{n} - S'(m_0)\frac{2}{n} - \frac{2S''(m_0)(m - m_0)}{n} + \frac{2S''(m_0)}{n^2}. \tag{62}$$

Now equating terms of order $\frac{1}{n^2}$ from (60) and (62) gives us the following relation for finding a,

$$a = -J - S''(m_0) \tag{63}$$

Substituting this back in the previous expression and matching terms of $1/n$ gives us the following expression for m_0

$$-Jm_0 + h - S'(m_0) = 0. \tag{64}$$

Now this above expression is precisely the first-order stationarity condition for the free energy of CW model. And from $a > 0$ we find that $\Phi''(m_0) = J - S''(m_0) > 0$ which tells us that m_0 lies at the minimum of the free energy.

This calculation establishes the following important observation: *Minima of the CW free energy defines distributions whose single-variable conditionals match those of the CW distribution*

Notice also that $\Phi(m)$ defined as in (3) is just the second order Taylor expansion of the free energy Ψ at m_0. Now starting from this observation we can define a class of potentially interesting metastable states by expanding further around the positive minima of Ψ.

$$\Phi^{(K)}(m) = \sum_{k=2}^{K} \Psi^{(k)}(m_0)\frac{(m - m_0)^k}{k!} \tag{65}$$

$$\nu^{(K)}(\underline{\sigma}) = \frac{e^{-n\Phi^{(K)}(m(\underline{\sigma}))}}{\binom{n}{\frac{n(1+m)}{2}}}. \tag{66}$$

A. Numerical evaluation of strong metastability violation in Curie-Weiss models

For distributions which are permuation invariant, i.e. where $\nu(\underline{\sigma})$ is only a function of $m(\underline{\sigma})$, the quantity in (3) can be computed efficiently my mapping the problem on to the magnetization space first.

First let us fix the Markov chain as Glauber dynamics defined by the Curie-Weiss model in (25). This gives us,

$$Pr(\underline{\sigma}_{\neg i}|\underline{\sigma}) = \frac{1}{n}\mu(-\sigma_i|\underline{\sigma}_{\setminus i}) \tag{67}$$

Now using this the violation in the detailed balance condition as defined in (3) can be rewritten as,

$$\frac{1}{2}\sum_{\underline{\sigma},\underline{\sigma}'}|P(\underline{\sigma}'|\underline{\sigma})\nu(\underline{\sigma}) - P(\underline{\sigma}|\underline{\sigma}')\nu(\underline{\sigma}')| = \frac{1}{2n}\sum_{i=1}^{n}\sum_{\underline{\sigma}}|\mu(-\sigma_i|\underline{\sigma}_{\setminus i})\nu(\underline{\sigma}) - \mu(\sigma_i|\underline{\sigma}_{\setminus i})\nu(\underline{\sigma}_{\neg i})| \tag{68}$$

$$= \frac{1}{2n}\sum_{i=1}^{n}\sum_{\underline{\sigma}}|(1-\mu(\sigma_i|\underline{\sigma}_{\setminus i}))\nu(\underline{\sigma}) - \mu(\sigma_i|\underline{\sigma}_{\setminus i})\nu(\underline{\sigma}_{\neg i})| \tag{69}$$

$$= \frac{1}{2n}\sum_{i=1}^{n}\sum_{\underline{\sigma}}|\nu(\underline{\sigma}) - \mu(\sigma_i|\underline{\sigma}_{\setminus i})(\nu(\underline{\sigma}_{\neg i}) + \nu(\underline{\sigma}))| \tag{70}$$

$$= \frac{1}{2n}\sum_{i=1}^{n}\sum_{\underline{\sigma}}\nu(\underline{\sigma}_{\setminus i})|\nu(\sigma_i|\underline{\sigma}_{\setminus i}) - \mu(\sigma_i|\underline{\sigma}_{\setminus i})| \tag{71}$$

Where $\nu(\underline{\sigma}_{\setminus i}) = \nu(\underline{\sigma}_{\neg i}) + \nu(\underline{\sigma})$ is the marginal distribution of all spins except σ_i

Now since $|\nu(\sigma_i|\underline{\sigma}_{\setminus i}) - \mu(\sigma_i|\underline{\sigma}_{\setminus i})| = |\nu(-\sigma_i|\underline{\sigma}_{\setminus i}) - \mu(-\sigma_i|\underline{\sigma}_{\setminus i})|$. We can deduce that $\sum_{\underline{\sigma}}\nu(\underline{\sigma}_{\setminus i})|\nu(\sigma_i|\underline{\sigma}_{\setminus i}) - \mu(\sigma_i|\underline{\sigma}_{\setminus i})| = \sum_{\underline{\sigma}}\nu(\underline{\sigma})|\nu(\sigma_i|\underline{\sigma}_{\setminus i}) - \mu(\sigma_i|\underline{\sigma}_{\setminus i})|$. This gives us the final expression,

$$\frac{1}{2}\sum_{\underline{\sigma},\underline{\sigma}'}|P(\underline{\sigma}'|\underline{\sigma})\nu(\underline{\sigma}) - P(\underline{\sigma}|\underline{\sigma}')\nu(\underline{\sigma}')| = \frac{1}{2n}\sum_{i=1}^{n}\sum_{\underline{\sigma}}\nu(\underline{\sigma})|\nu(\sigma_i|\underline{\sigma}_{\setminus i}) - \mu(\sigma_i|\underline{\sigma}_{\setminus i})| \tag{72}$$

For the Curie-Weiss model, we can easily see that the single-variable conditionals have the following expression that depends only on σ_i and the magnetization of the state,

$$\mu(\sigma_i|\underline{\sigma}_{\setminus i}) = \frac{1}{2}(1-\tanh(\sigma_i(Jm(\underline{\sigma})-h)+\frac{J}{n}) \equiv f(\sigma_i, m(\underline{\sigma})). \tag{73}$$

Now we assume a general permutation invariant form for ν,

$$\nu(\underline{\sigma}) = \frac{e^{-n(\Phi(m(\underline{\sigma}))+S(m(\underline{\sigma})))}}{Z_\Phi} \equiv \frac{e^{-nH(m(\underline{\sigma}))}}{Z_\Phi} \tag{74}$$

From this we can get the conditional as,

$$\nu(\sigma_i|\underline{\sigma}_{\setminus i}) = \frac{1}{1+\frac{\nu(\underline{\sigma}_{\neg i})}{\nu(\underline{\sigma})}} = \frac{1}{1+\exp(n(H(m(\underline{\sigma}) - H(m(\underline{\sigma}_{\neg i})))} \equiv g(\sigma_i, m(\underline{\sigma})) \tag{75}$$

We can use these expressions for conditionals in (72) and write this completely in the magnetization space. The number of states with a fixed per spin magnetization m is simply given by $\exp(nS(m))$. Now for a given i, a $\frac{1+m}{2}$

fraction of these will have $\sigma_i = 1$. Using these we can write,

$$\frac{1}{2n}\sum_{i=1}\sum_{\underline{\sigma}}\nu(\underline{\sigma})|\nu(\sigma_i|\underline{\sigma}_{\setminus i})-\mu(\sigma_i|\underline{\sigma}_{\setminus i})| \tag{76}$$

$$=\frac{1}{2Z_\Phi n}\sum_{i=1}^{n}\sum_{m\in\{-1,-1+\frac{2}{n},\dots,1\}}e^{-n\Phi(m)}\left(\frac{1+m}{2}|g(1,m)-f(1,m)|+\frac{1-m}{2}|g(-1,m)-f(-1,m)|\right), \tag{77}$$

$$=\frac{1}{2Z_\Phi}\sum_{m\in\{-1,-1+\frac{2}{n},\dots,1\}}e^{-n\Phi(m)}|g(1,m)-f(1,m)| \tag{78}$$

Where in the last line we have use the normalization condition on the conditionals, $g(1,m)+g(-1,m) = f(1,m)+f(-1,m) = 1$. Using this reformulation we can compute the strong metastability of the state in essentially $O(n)$ time. The normalization constant can also be computed efficiently using the formula, $Z_\phi = \sum_\sigma \exp(-nH(m(\underline{\sigma}))) = \sum_m \exp(-n\Phi(m))$.

The details of numerical evaluation of three different families metastable distributions for the CW model is shown in FIG.2. First we look at the quartic ($K = 4$) and quadratic ($K = 2$) approximations to the CW free energy as defined in (66). We perform the expansion around the positive minimum of the CW free energy ($m_0 > 0$). As this corresponds to the mode of the distribution with suppressed probability when $h > 0$ (refer FIG.3b). For the third case, we truncate the free energy around m_0 with a certain width,

$$\Phi(m) = \begin{cases} \Psi(m), & \text{if } |m-m_0| < \frac{m_0}{4\sqrt{a}} \\ \infty, & \text{otherwise} \end{cases} \tag{79}$$

This truncation corresponds to the type of metastable states defined in Section II B. We see numerically that these metastable states have an η value that is exponentially small in the system size.

SUPPLEMENTARY NOTE 4: ROBUSTNESS OF STRONG METASTABILITY: PROOFS

A. Proof of Proposition 2

Proof.

$$\frac{1}{2}\sum_{i,j\in\mathcal{S}}|P(i|j)\nu(j)-P(j|i)\nu(i)| = \frac{1}{2}\sum_{i,j\in\mathcal{S}}|\sum_k p_k(P(i|j)\nu_k(j)-P(j|i)\nu_k(i))|, \tag{80}$$

$$\le \frac{1}{2}\sum_{i,j\in\mathcal{S}}\sum_k p_k|(P(i|j)\nu_k(j)-P(j|i)\nu_k(i))| \le \sum_k p_k\eta_k. \tag{81}$$

□

B. Proof of Proposition 3

Proof. Fix an integer $t \ge 1$, and define $\nu' = \nu P^t$. Also define $\Delta(i) \coloneqq \nu'(i) - \nu(i)$. We bound the detailed-balance violation of ν' as

$$\frac{1}{2}\sum_{i,j\in\mathcal{S}}|P(i|j)\nu'(j)-P(j|i)\nu'(i)| \le \frac{1}{2}\sum_{i,j\in\mathcal{S}}|P(i|j)\nu(j)-P(j|i)\nu(i)|+|P(i|j)\Delta(j)-P(j|i)\Delta(i)| \tag{82}$$

$$\le \eta + \frac{1}{2}\sum_j\left(\sum_i P(i|j)\right)|\Delta(j)| + \frac{1}{2}\sum_i\left(\sum_j P(j|i)\right)|\Delta(i)| \tag{83}$$

$$\le \eta + \|\Delta\|_1 = \eta + 2|\nu - \nu P^t|_{TV}. \tag{84}$$

Now via triangle inequality,

$$|\nu - \nu P^t|_{TV} \leq |\nu - \nu P|_{TV} + |\nu P - \nu P^2|_{TV} + \ldots + |\nu P^{t-1} - \nu P^t|_{TV}. \tag{85}$$

Since strong metastability implies metastability, ν is η-metastable. By the data processing inequality, νP^t is also η-metastable, as are the intermediate states.

$$|\nu - \nu P^t|_{TV} \leq t\eta, \tag{86}$$

and substituting above yields

$$\frac{1}{2}\sum_{i,j\in\mathcal{S}} |P(i|j)\nu'(j) - P(j|i)\nu'(i)| \leq (1+2t)\eta. \tag{87}$$

□

SUPPLEMENTARY NOTE 5: PROOF OF THEOREM 1

Proof. The $\eta-$strong metastability condition gives us,

$$\frac{1}{2}\sum_{\underline{\sigma},\underline{\sigma}'\in\mathcal{S}} |P(\underline{\sigma}'|\underline{\sigma})\nu(\underline{\sigma}) - P(\underline{\sigma}|\underline{\sigma}')\nu(\underline{\sigma}')| \leq \eta \tag{88}$$

Now let us introduce the notation $\underline{\sigma}_{i\to\alpha}$ to represent $\underline{\sigma}$ with the i-th variable replaced by the alphabet α.

Now if we consider only one variable transitions of the chain we get,

$$\frac{1}{2}\sum_{\underline{\sigma}\in\mathcal{S}}\sum_{u=1}^{n}\sum_{\alpha\in Q,\alpha\neq\sigma_i} |P(\underline{\sigma}_{u\to\alpha}|\underline{\sigma})\nu(\underline{\sigma}) - P(\underline{\sigma}|\underline{\sigma}_{u\to\alpha})\nu(\underline{\sigma}_{u\to\alpha})| \leq \eta \tag{89}$$

Since P is a reversible Markov chain, the following relations can be derived from the detailed balance conditions,

$$\frac{P(\underline{\sigma}_{u\to\alpha}|\underline{\sigma})}{P(\underline{\sigma}|\underline{\sigma}_{u\to\alpha})} = \frac{\mu(\underline{\sigma}_{u\to\alpha})}{\mu(\underline{\sigma})} = \frac{\mu(\alpha|\underline{\sigma}_{\setminus u})}{\mu(\sigma_u|\underline{\sigma}_{\setminus u})}. \tag{90}$$

After plugging these into (89) and a bit of algebra gives us,

$$\frac{1}{2}\sum_{\underline{\sigma}\in\mathcal{S}}\sum_{u=1}^{n}\sum_{\alpha\in Q,\alpha\neq\sigma_i} \frac{P(\underline{\sigma}_{u\to\alpha}|\underline{\sigma})}{\mu(\alpha|\underline{\sigma}_{\setminus u})}\Big|\mu(\alpha|\underline{\sigma}_{\setminus u})\nu(\underline{\sigma}) - \mu(\sigma_u|\underline{\sigma}_{\setminus u})\nu(\underline{\sigma}_{u\to\alpha})\Big| \leq \eta. \tag{91}$$

Now we can take the marginal probability $\nu(\underline{\sigma}_{\setminus u})$ out of the expression within the summations in the LHS. Then we can use the definition of conditionals given by: $\nu(\sigma_i|\sigma_{\setminus i}) = \frac{\nu(\underline{\sigma})}{\nu(\underline{\sigma}_{\setminus i})}$, $\nu(\alpha|\sigma_{\setminus i}) = \frac{\nu(\underline{\sigma}_{u\to\alpha})}{\nu(\underline{\sigma}_{\setminus i})}$, to rewrite it as follows,

$$\frac{1}{2}\sum_{\underline{\sigma}\in\mathcal{S}}\sum_{u=1}^{n}\sum_{\alpha\in Q,\alpha\neq\sigma_i} \frac{P(\underline{\sigma}_{u\to\alpha}|\underline{\sigma})}{\mu(\alpha|\underline{\sigma}_{\setminus u})}\nu(\underline{\sigma}_{\setminus u})\Big|\mu(\alpha|\underline{\sigma}_{\setminus u})\nu(\sigma_u|\underline{\sigma}_{\setminus u}) - \mu(\sigma_u|\underline{\sigma}_{\setminus u})\nu(\alpha|\underline{\sigma}_{\setminus u})\Big| \leq \eta. \tag{92}$$

Now applying Condition 1 we can write this as,

$$\frac{1}{2}\sum_{\underline{\sigma}\in\mathcal{S}}\sum_{u=1}^{n}\nu(\underline{\sigma}_{\setminus u})\sum_{\alpha\in Q,\alpha\neq\sigma_i}\Big|\mu(\alpha|\underline{\sigma}_{\setminus u})\nu(\sigma_u|\underline{\sigma}_{\setminus u})-\mu(\sigma_u|\underline{\sigma}_{\setminus u})\nu(\alpha|\underline{\sigma}_{\setminus u})\Big|\leq\frac{\eta}{\omega_P}. \tag{93}$$

Now we assert that the expression $\sum_{\alpha\in[Q],\alpha\neq\sigma_i}\Big|\mu(\alpha|\underline{\sigma}_{\setminus u})\nu(\sigma_u|\underline{\sigma}_{\setminus u})-\mu(\sigma_u|\underline{\sigma}_{\setminus u})\nu(\alpha|\underline{\sigma}_{\setminus u})\Big|$ is lower bounded simply by $|\nu(\sigma_u|\underline{\sigma}_{\setminus u})-\mu(\sigma_u|\underline{\sigma}_{\setminus u})|$.

To show this consider two vectors $a, b\in\mathbb{R}^{|Q|}$, such that they sum to one, $\sum_i a_i=\sum_i b_i=1$. It is simple to see that,

$$|a_k-b_k|=|a_k\sum_{j\in Q}b_j-b_k\sum_{j\in Q}a_j|=|\sum_{j\in Q,j\neq k}a_kb_j-a_jb_k|\leq\sum_{j\in Q,j\neq k}|a_kb_j-a_jb_k| \tag{94}$$

Since the conditionals sum to one, as a consequence of the above inequality we can see that, $\sum_{\alpha\in Q,\alpha\neq\sigma_i}\Big|\mu(\alpha|\underline{\sigma}_{\setminus u})\nu(\sigma_u|\underline{\sigma}_{\setminus u})-\mu(\sigma_u|\underline{\sigma}_{\setminus u})\nu(\alpha|\underline{\sigma}_{\setminus u})\Big|\geq|\nu(\sigma_u|\underline{\sigma}_{\setminus u})-\mu(\sigma_u|\underline{\sigma}_{\setminus u})|$. Putting this into (93), we get,

$$\frac{1}{2}\sum_{u=1}^{n}\sum_{\underline{\sigma}\in\mathcal{S}}\nu(\underline{\sigma}_{\setminus u})\Big|\nu(\sigma_u|\underline{\sigma}_{\setminus u})-\mu(\sigma_u|\underline{\sigma}_{\setminus u})\Big|\leq\frac{\eta}{\omega_P}. \tag{95}$$

Now by splitting the inner sum over $\underline{\sigma}$ as one going over σ_u and another over $\underline{\sigma}_{\setminus u}$, we can easily deduce that,

$$\sum_{u=1}^{n}\sum_{\underline{\sigma}\in\mathcal{S}}\nu(\underline{\sigma})\Big|\nu(.|\underline{\sigma}_{\setminus u})-\mu(.|\underline{\sigma}_{\setminus u}))\Big|_{TV}\leq\frac{\eta}{\omega_P}. \tag{96}$$

□

SUPPLEMENTARY NOTE 6: PROOF OF THEOREM 2

Proof. For a fixed $u\in[n]$ and parametric conditionals are given by $p(q|\underline{\sigma}_{\setminus u};\underline{\theta})=\frac{1}{1+\sum_{p\in Q,p\neq q}e^{E(\underline{\sigma}_{u\to p},\underline{\theta})-E(\underline{\sigma}_{u\to q},\underline{\theta})}}$. And the corresponding log-likelihoods are $\mathcal{L}_u(\underline{\theta},\underline{\sigma})=-\log(p(\sigma_u|\underline{\sigma}_{\setminus u};\underline{\theta}))$ The average KL divergence between the conditionals of ν and the parametric hypothesis can be written as,

$$\mathop{\mathbb{E}}_{\underline{\sigma}_{\setminus u}\sim\nu_{\setminus u}}D_{KL}\left(\nu(.|\underline{\sigma}_{\setminus u}||p(.|\underline{\sigma}_{\setminus u};\underline{\theta}_u)\right)=\mathop{\mathbb{E}}_{\underline{\sigma}\sim\nu}\log\left(\nu(\sigma_u|\underline{\sigma}_{\setminus u})(1+\sum_{p\in Q,p\neq q}e^{E(\underline{\sigma}_{u\to p},\underline{\theta})-E(\underline{\sigma}_{u\to q},\underline{\theta})})\right) \tag{97}$$

$$=\mathop{\mathbb{E}}_{\underline{\sigma}\sim\nu}\log\left(\nu(\sigma_u|\underline{\sigma}_{\setminus u})\right)+\mathop{\mathbb{E}}_{\underline{\sigma}\sim\nu}\mathcal{L}_u(\underline{\theta},\underline{\sigma}) \tag{98}$$

For convenience, let us define ρ as the lower bound on the conditionals of the parametric distribution, which is assumed to also include the true distribution, i.e. $\rho\equiv\min_{\sigma,\underline{\theta},u}p(\sigma_u|\underline{\sigma}_{\setminus u};\underline{\theta})$.

From the reverse Pinsker's inequality (Lemma 4.1 in [6]) and the lower bound on the conditionals we see that, $D_{KL}(\nu(.|\sigma_{\setminus u})||\mu(.|\sigma_{\setminus u}))\leq\frac{2}{\rho}\Big|\nu(.|\underline{\sigma}_{\setminus u})-\mu(.|\underline{\sigma}_{\setminus u})\Big|_{TV}$. Using this bound in Theorem 1 gives us,

$$\mathop{\mathbb{E}}_{\underline{\sigma}\sim\nu}\log(\nu(\sigma_u|\sigma_{\setminus u}))+\mathop{\mathbb{E}}_{\underline{\sigma}\sim\nu}\mathcal{L}(\underline{\theta}^*_u,\underline{\sigma})\leq\frac{2\eta}{\rho\ \omega_P}. \tag{99}$$

Now from the positivity of KL divergence, $\mathbb{E}_{\underline{\sigma}\sim\nu} \log(\nu(\sigma_u|\sigma_{\setminus u})) \geq \mathbb{E}_{\underline{\sigma}\sim\nu} \log(p(\sigma_u|\sigma_{\setminus u}; \hat{\underline{\theta}}_u)) = -\mathbb{E}_{\underline{\sigma}\sim\nu} \mathcal{L}(\hat{\underline{\theta}}_u, \underline{\sigma})$. This gives us,

$$\mathbb{E}_{\underline{\sigma}\sim\nu} \mathcal{L}(\underline{\theta}^*_u, \underline{\sigma}) - \mathbb{E}_{\underline{\sigma}\sim\nu} \mathcal{L}(\hat{\underline{\theta}}_u, \underline{\sigma}) \leq \frac{2\eta}{\rho\ \omega_P} \tag{100}$$

Now the bounds on the parametric conditionals can be used to bound the likelihood $\mathcal{L}(\theta, \underline{\sigma})$ as follows, $\log(\frac{1}{1-\rho}) \leq \mathcal{L}(\theta, \underline{\sigma}) \leq \log(\frac{1}{\rho})$. This establishes $\mathcal{L}(\theta, \sigma)$ as a bounded random variable. Now we can use Hoeffding's inequality (Lemma eflem:hoeff) to show that with probability $1-\delta$,

$$\frac{1}{M'}\left(\sum_{t=1}^{M'} \mathcal{L}(\underline{\theta}^*_u, \underline{\sigma}^{(t)}) - \sum_{t=1}^{M'} \mathcal{L}(\hat{\underline{\theta}}_u, \underline{\sigma}^{(t)})\right) \leq \mathbb{E}_{\underline{\sigma}\sim\nu} \mathcal{L}(\underline{\theta}^*_u, \underline{\sigma}) - \mathbb{E}_{\underline{\sigma}\sim\nu} \mathcal{L}(\hat{\underline{\theta}}_u, \underline{\sigma}) + \log\left(\frac{1-\rho}{\rho}\right)\sqrt{\frac{\log(1/\delta)}{2M'}}, \tag{101}$$

$$\leq \frac{2\eta}{\rho\omega_P} + \log\left(\frac{1-\rho}{\rho}\right)\sqrt{\frac{\log(1/\delta)}{2M'}}. \tag{102}$$

Now from (16) we see that $\rho \geq \frac{1}{1+(|Q|-1)e^{2\gamma}}$. Plugging this into the above equation gives us the desired result.

□

SUPPLEMENTARY NOTE 7: PROOFS OF LEARNING GUARANTEES FOR ISING MODELS

For Ising models, the exact PL estimator and the one computed from samples have the following form

$$\mathcal{L}(\underline{\theta}_u) = \mathbb{E}_{\underline{\sigma}\sim\nu} \mathcal{L}(\underline{\theta}_u, \underline{\sigma}) = \mathbb{E}_{\underline{\sigma}\sim\nu} \log(1 + \exp(-2(\sum_{k\neq u} \theta_{u,k}\sigma_u\sigma_k + \theta_u))) \tag{103}$$

$$\mathcal{L}_M(\underline{\theta}_u) = \frac{1}{M}\sum_{t=1}^{M} \mathcal{L}(\underline{\theta}_u, \underline{\sigma}^{(t)}) = \frac{1}{M}\sum_{t=l}^{M} \log(1 + \exp(-2\sigma_u^{(t)}(\sum_{k\neq u} \theta_{u,k}\sigma_k^{(t)} + \theta_u))) \tag{104}$$

$$\hat{\underline{\theta}}_u \coloneqq \underset{||\underline{\theta}_u||_1 \leq \gamma}{\operatorname{argmin}} \mathcal{L}_M(\underline{\theta}_u). \tag{105}$$

A key quantity we will use through out this proof is the following measure of curvature of the loss around $\underline{\theta}^*_u$

$$\delta\mathcal{L}_M(\Delta, \underline{\theta}^*_u) \coloneqq \mathcal{L}_M(\hat{\underline{\theta}}_u) - \mathcal{L}_M(\underline{\theta}^*_u) - \langle \Delta, \nabla\mathcal{L}_M(\underline{\theta}^*_u)\rangle \tag{106}$$

1. *Proof of Theorem 3*

Proof. First let us define two error vectors that respectively capture the error in all parameters connected to a variable at u and only the error in the pairwise terms connected to u.

$$\Delta \coloneqq \hat{\underline{\theta}}_u - \underline{\theta}^*_u \in \mathbb{R}^n, \tag{107}$$

$$\Delta_{\setminus u} \coloneqq \hat{\underline{\theta}}_{\setminus u} - \underline{\theta}^*_{\setminus u} \in \mathbb{R}^{n-1}. \tag{108}$$

That is, these two error vectors only differ by $\hat{\theta}_u - \theta^*_u$.

Now from the definition of the curvature function in (106),

$$\mathcal{L}_M(\underline{\hat{\theta}}_u) - \mathcal{L}_M(\underline{\theta}^*_u) = \delta\mathcal{L}_M(\Delta, \underline{\theta}^*_u) + \langle \Delta, \nabla\mathcal{L}_M(\underline{\theta}^*_u)\rangle, \tag{109}$$

$$\geq \delta\mathcal{L}_M(\Delta, \underline{\theta}^*_u) - |\langle \Delta, \nabla\mathcal{L}_M(\underline{\theta}^*_u)\rangle|, \tag{110}$$

$$\geq \delta\mathcal{L}_M(\Delta, \underline{\theta}^*_u) - ||\Delta||_1 ||\nabla\mathcal{L}_M(\underline{\theta}^*_u)||_\infty \tag{111}$$

Now to avoid a contradiction with the definition of $\underline{\hat{\theta}}$ as the minimizer of $\mathcal{L}_M$, the quantity on the left must be negative. This then implies that $\delta\mathcal{L}_M(\Delta, \underline{\theta}^*_u) \leq ||\Delta||_1 ||\nabla\mathcal{L}_M(\underline{\theta}^*_u)||_\infty$. Due to the ℓ_1 constraint in the problem we have $||\Delta||_1 \leq 2\gamma$. This gives us,

$$\delta\mathcal{L}_M(\Delta, \underline{\theta}^*_u) \leq 2\gamma ||\nabla\mathcal{L}_M(\underline{\theta}^*_u)||_\infty. \tag{112}$$

Now we have two technical lemmas that give relevant bounds that can turn the above inequality in to a learning guarantee.

From Lemma 3, when $M \geq \frac{8}{\varepsilon_a^2}\log(\frac{4n^2}{\delta})$ the following statement holds with probability $1 - \frac{\delta}{2n}$,

$$||\nabla\mathcal{L}_M(\underline{\theta}^*_u)||_\infty \leq \frac{4\eta}{\omega_P} + \varepsilon_a \tag{113}$$

From Lemma 6, when $M \geq \frac{2\gamma^4}{\varepsilon_b^2}\log(\frac{2n}{\delta})$, the following statement holds with probability $1 - \frac{\delta}{2n}$,

$$\delta\mathcal{L}_M(\Delta, \underline{\theta}^*_u) \geq \frac{e^{-4\gamma}}{2}||\Delta_{\setminus u}||^2_\infty - \frac{4e^{-2\gamma}\eta}{\omega_P} - \varepsilon_b \tag{114}$$

Using these bounds in (112) gives us the following bound with probability $1 - \delta/n$,

$$||\Delta_{\setminus u}||^2_\infty - \frac{8\eta e^{2\gamma}}{\omega_P} - 2e^{4\gamma}\varepsilon_b \;\leq\; 4e^{4\gamma}\gamma\left(\frac{4\eta}{\omega_P} + \varepsilon_a\right). \tag{115}$$

Now make the following choices; choose ε_a and ε_b such that $4e^{4\gamma}\gamma\varepsilon_a = 2e^{4\gamma}\varepsilon_b = \varepsilon^2/2$, and choose $M = \left\lceil 2^9\frac{e^{8\gamma}\gamma^4}{\varepsilon^4}\log(\frac{8n^2}{\delta})\right\rceil \geq \max\left(\frac{2\gamma^4}{\varepsilon_b^2}\log(\frac{2n}{\delta}), \frac{8}{\varepsilon_a^2}\log(\frac{8n^2}{\delta})\right)$. Plugging these choices in the expression above gives us the following bound with probability $1 - \delta/n$.

$$||\Delta_{\setminus u}||^2_\infty \leq \frac{8\eta e^{2\gamma}}{\omega_P} + \frac{16e^{4\gamma}\gamma\eta}{\omega_P} + \varepsilon^2, \tag{116}$$

$$\leq \frac{16\eta(1+\gamma)e^{4\gamma}}{\omega_P} + \varepsilon^2. \tag{117}$$

$$\tag{118}$$

This implies that with the same probability,

$$||\underline{\theta}^*_{\setminus u} - \underline{\hat{\theta}}_{\setminus u}||_\infty \leq \sqrt{\varepsilon^2 + \frac{16\eta(1+\gamma)e^{4\gamma}}{\omega_P}} \leq \;\varepsilon + 4e^{2\gamma}\sqrt{\frac{(1+\gamma)\eta}{\omega_P}} \tag{119}$$

Now a union bound over each $u \in [n]$ gives us the desired bound in the theorem statement.

□

A. Technical lemmas for PL estimator without sparsity

1. Gradient concentration

Lemma 2. For an η-strongly metastable $||\nabla\mathcal{L}(\underline{\theta}_u^*)||_\infty \leq \frac{4\eta}{\omega_P}$

Proof.

$$\frac{\partial \mathcal{L}(\underline{\theta}_u^*)}{\partial \theta_{uk}} = \mathop{\mathbb{E}}_{\underline{\sigma}\sim\nu} \frac{-2\sigma_u\sigma_k}{1+\exp(2\sigma_u(\sum_{k'\neq u}\theta^*_{u,k'}\sigma_{k'}+\theta^*_u))}, \tag{120}$$

$$= -2\mathop{\mathbb{E}}_{\underline{\sigma}\sim\nu} \mu(-\sigma_u|\sigma_{\setminus u})\sigma_u\sigma_k, \tag{121}$$

$$= -2\mathop{\mathbb{E}}_{\underline{\sigma}\sim\nu} \left(\mu(-\sigma_u|\sigma_{\setminus u}) - \nu(-\sigma_u|\sigma_{\setminus u})\right)\sigma_k\sigma_u. \tag{122}$$

In the last line, we have used following relation that holds for all $k\neq u$,

$$\mathop{\mathbb{E}}_{\underline{\sigma}\sim\nu}\nu(-\sigma_u|\sigma_{\setminus u})\sigma_u\sigma_k = \sum_{\underline{\sigma}}\nu(\underline{\sigma}_{\setminus u})\nu(\sigma_u|\underline{\sigma}_{\setminus u})\nu(-\sigma_u|\underline{\sigma}_{\setminus u})\sigma_u\sigma_k = \sum_{\underline{\sigma}_{\setminus u}}\nu(\underline{\sigma}_{\setminus u})\underbrace{\nu(\sigma_u|\underline{\sigma}_{\setminus u})\nu(-\sigma_u|\underline{\sigma}_{\setminus u})}_{\text{this is independent of }\sigma_u.}\sigma_k\sum_u\sigma_u = 0.$$

Now we can bound this gradient directly from Theorem 1,

$$\left|\frac{\partial\mathcal{L}(\underline{\theta}_u^*)}{\partial\theta_{u,k}}\right| \leq 2\mathop{\mathbb{E}}_{\underline{\sigma}\sim\nu}\left|\left(\mu(-\sigma_u|\sigma_{\setminus u}) - \nu(-\sigma_u|\sigma_{\setminus u})\right)\right| \tag{123}$$

$$\leq \frac{4\eta}{\omega_P}. \tag{124}$$

Similarly one can also show that,

$$\left|\frac{\partial\mathcal{L}(\underline{\theta}_u^*)}{\partial\theta_u}\right| \leq \frac{4\eta}{\omega_P}. \tag{125}$$

□

Lemma 3. For a $\eta-$strongly metastable distribution and $\varepsilon_a,\delta_a>0$. Given $M\geq\frac{8}{\varepsilon_a^2}\log(\frac{2n}{\delta_a})$ i.i.d. samples guarantees with probability at least $1-\delta_a$ that,

$$||\nabla\mathcal{L}_M(\underline{\theta}_u^*)||_\infty \leq \frac{4\eta}{\omega_P}+\varepsilon_a \tag{126}$$

Proof. By direct calculation as done in the proof of Lemma 2,

$$\frac{\partial\mathcal{L}(\underline{\theta}_u^*)}{\partial\theta_{u,k}} = -2\mathop{\mathbb{E}}_{\underline{\sigma}\sim\nu}\mu(-\sigma_u|\sigma_{\setminus u})\sigma_u\sigma_k. \tag{127}$$

Now the variable in the expectation can be easily bounded,

$$-1\leq\sigma_u\sigma_k\mu(-\sigma_u|\underline{\sigma}_{\setminus u})\leq 1. \tag{128}$$

This implies from Hoeffding's inequality that,

$$Pr\left(\left|\frac{\partial\mathcal{L}(\underline{\theta}_u^*)}{\partial\theta_{u,k}} - \frac{\partial\mathcal{L}_M(\underline{\theta}_u^*)}{\partial\theta_{u,k}}\right|\geq\varepsilon_a\right)\leq 2\exp\left(\frac{-M\varepsilon_a^2}{8}\right). \tag{129}$$

Repeating the same steps we can show that a similar tail bound also holds for the derivative w.r.t θ_u. Now choosing $M \geq \frac{8}{\varepsilon_a^2}\log(\frac{2n}{\delta_a})$ gives us the desired results by the union bound.

□

2. *Strong convexity-bounds on curvature*

It is useful to define the following function on reals,

$$f(x) \coloneqq \log(1+\exp(-2x)). \tag{130}$$

Lemma 4. (Smoothnes and Strong convexity of f)
Let f be as defined in (130), and let $\delta f(x,\varepsilon) \coloneqq f(x+\varepsilon) - (f(x)+\varepsilon f'(x))$. Then if $\max(|x|,|x+\varepsilon|) \leq \gamma$,

$$\frac{\varepsilon^2}{2} \geq \delta f(x,\varepsilon) \geq \frac{\exp(-2\gamma)\varepsilon^2}{2} \tag{131}$$

Proof. This lemma can be shown using standard arguments from the strong convexity of f [7]. We can see that for any $|y| \leq \gamma$ we have,

$$f''(y) = \frac{2}{1+\cosh(2y)} \geq \frac{2}{1+\cosh(2\gamma)} \geq \exp(-2\gamma). \tag{132}$$

From this, it is clear that f is convex in the domain $[-\gamma,\gamma]$. Moreover, the above expression gives that, $g(y) \coloneqq f(y) - \frac{exp(-2\gamma)}{2}y^2$ is also a convex function in the same domain. Now from the first-order convexity condition, $g(x+\varepsilon) \geq g(x) + \varepsilon g'(x)$, we can find the lower bound on δf.

Similarly, we can see that $f''(y) < 1$. This implies that $h(y) \coloneqq \frac{y^2}{2} - f(y)$ is also a convex function. From the relation $h(x+\varepsilon) \geq h(x) + \varepsilon h'(x)$ we get the upper bound on the δf. □

Lemma 5. $\delta\mathcal{L}(\Delta,\underline{\theta}_u^*) \geq \frac{e^{-4\gamma}}{2}||\Delta_{\setminus u}||_\infty^2 - \frac{8e^{-2\gamma}\eta}{\omega_P}$

Proof. Define local energy function $E_u(\underline{\sigma};\underline{\theta}) = \sum_{k\neq u}\theta_{u,k}\sigma_u\sigma_k + \theta_u\sigma_u$. Notice that this function is linear in the θ variables. Now PL loss can be expressed as a function of this local energy. From the definition of f in (130), we have the following relations,

$$\mathcal{L}(\underline{\theta}_u + \Delta) = \mathop{\mathbb{E}}_{\underline{\sigma}\sim\nu} f(E_u(\underline{\sigma};\underline{\theta}_u+\Delta)) = \mathop{\mathbb{E}}_{\underline{\sigma}\sim\nu} f(E_u(\underline{\sigma};\underline{\theta}_u) + E_u(\underline{\sigma};\Delta)). \tag{133}$$

$$\langle\nabla\mathcal{L}(\underline{\theta}_u),\Delta\rangle = \mathop{\mathbb{E}}_{\underline{\sigma}\sim\nu} f'(E_u(\underline{\sigma};\underline{\theta}_u))(\sum_{k\neq u}\frac{\partial E_u(\underline{\sigma};\underline{\theta}_u)}{\partial\theta_{uk}}\Delta_{uk} + \frac{\partial E_u(\underline{\sigma};\underline{\theta}_u)}{\partial\theta_u}\Delta_u) = \mathop{\mathbb{E}}_{\underline{\sigma}\sim\nu} f'(E_u(\underline{\sigma};\underline{\theta}_u))E_u(\underline{\sigma};\Delta). \tag{134}$$

Now from the definitions in Lemma 4, we can define the curvature of $\mathcal{L}$ mimicking (106) as follows,

$$\delta\mathcal{L}(\Delta,\underline{\theta}_u^*) = \mathop{\mathbb{E}}_{\underline{\sigma}\sim\nu}\delta f(E_u(\underline{\sigma},\underline{\theta}_u^*),E_u(\underline{\sigma},\Delta)). \tag{135}$$

Now from Condition 2 we have, $|E_u(\underline{\sigma},\underline{\theta}_u^*)| \leq ||\underline{\theta}_u^*||_1 \leq \gamma$. The same condition will also hold for $|E_u(\underline{\sigma},\hat{\underline{\theta}}_u)|_1$ as these constraints are imposed in the optimization.

This implies from Lemma 4 that,

$$\delta\mathcal{L}(\Delta,\underline{\theta}_u^*) \geq \frac{\exp(-2\gamma)}{2}\mathop{\mathbb{E}}_{\underline{\sigma}\sim\nu}(E_u(\underline{\sigma},\Delta))^2 = \frac{\exp(-2\gamma)}{2}\mathop{\mathbb{E}}_{\underline{\sigma}\sim\nu}\left(\sum_{k\neq u}\sigma_k\Delta_{u,k}+\Delta_u\right) \geq \frac{\exp(-2\gamma)}{2}\mathop{\mathrm{Var}}_{\underline{\sigma}\sim\nu}\left[\sum_{k\neq u}\Delta_{uk}\sigma_k\right]. \tag{136}$$

Now for some $i \neq u$, we can use the law of conditional variances to bound this quantity. The choice of i will be made later to get optimal bounds.

$$\mathop{\mathrm{Var}}_{\underline{\sigma}\sim\nu}\left[\sum_{k\neq u}\Delta_{uk}\sigma_k\right] \geq \mathop{\mathbb{E}}_{\underline{\sigma}_{\setminus i}\sim\nu_{\setminus i}}\mathop{\mathrm{Var}}_{\sigma_i\sim\nu(.|\underline{\sigma}_{\setminus i})}\left(\sum_{k\neq u}\Delta_{uk}\sigma_k\middle|\underline{\sigma}_{\setminus i}\right) = \Delta_{ui}^2\mathop{\mathbb{E}}_{\underline{\sigma}_{\setminus i}\sim\nu_{\setminus i}}\mathop{\mathrm{Var}}_{\sigma_i\sim\nu(.|\underline{\sigma}_{\setminus i})}\left(\sigma_k\middle|\underline{\sigma}_{\setminus i}\right) \tag{137}$$

Now as a consequence of the closeness of conditionals proved in Theorem 1, we can show that the conditional variance of the metastable state is close to that of the true distribution μ. This is shown in Lemma 7. Moreover the conditional variance w.r.t μ is naturally lower bounded by the finite temperature bound,

$$\mathop{\mathrm{Var}}_{\sigma_u\sim\mu(.|\underline{\sigma}_{\setminus u})}[\sigma_u|\underline{\sigma}_{\setminus u}] = 1-\tanh^2(\sum_{j\neq u}\theta_{uj}^*\sigma_j+\theta_u^*) \geq 1-\tanh^2(\gamma) \geq e^{-2\gamma}. \tag{138}$$

Now define a function that captures the error incurred in replacing the metastable distribution with the equilibrium distribution in the conditional variance,
$G(\underline{\sigma}_{\setminus i}) := \mathop{\mathrm{Var}}_{\sigma_i\sim\nu(.|\underline{\sigma}_{\setminus i})}\left[\sigma_i|\underline{\sigma}_{\setminus i}\right] - \mathop{\mathrm{Var}}_{\sigma_i\sim\mu(.|\underline{\sigma}_{\setminus i})}\left[\sigma_i|\underline{\sigma}_{\setminus i}\right]$

Using this in (137) we find,

$$\mathop{\mathrm{Var}}_{\underline{\sigma}\sim\nu}\left[\sum_{k\neq u}\Delta_{uk}\sigma_k\right] \geq \Delta_{ui}^2\left(e^{-2\gamma}-\mathop{\mathbb{E}}_{\underline{\sigma}\sim\nu}|G(\underline{\sigma}_{\setminus i})|\right) \tag{139}$$

$$\tag{140}$$

Now by using the result in Lemma 7 we can upper bound $\mathop{\mathbb{E}}_{\underline{\sigma}\sim\nu}|G(\underline{\sigma}_{\setminus i})|$. This gives us

$$\mathop{\mathrm{Var}}_{\underline{\sigma}\sim\nu}\left[\sum_{k\neq u}\Delta_{uk}\sigma_k\right] \geq \Delta_{ui}^2\left(e^{-2\gamma}-\frac{4\eta}{\omega_P}\right) = ||\Delta_{\setminus u}||_\infty^2\left(e^{-2\gamma}-\frac{4\eta}{\omega_P}\right) \tag{141}$$

$$\tag{142}$$

We have chosen the i here such that $\Delta_{ui}^2 = ||\Delta_{\setminus u}||_\infty^2$. Plugging this back in (136) gives us the desired result.

□

Lemma 6. For a $\eta-$strongly metastable distribution and $\varepsilon_b, \delta_a \geq 0$. Given $M \geq \frac{2\gamma^4}{\varepsilon_b^2}\log(\frac{1}{\delta_b})$ guarantees with probability at least $1-\delta_b$ that, $\delta\mathcal{L}_M(\Delta,\underline{\theta}_u^*) \geq \frac{e^{-4\gamma}}{2}||\Delta||_\infty^2 - \frac{4e^{-2\gamma}\eta}{\omega_P} - \varepsilon_b$

Proof.

$$\delta\mathcal{L}_M(\Delta,\underline{\theta}^*) = \frac{1}{M}\sum_{t=1}^{M}\delta f(E_u(\underline{\sigma}^{(t)},\underline{\theta}^*),E_u(\underline{\sigma}^{(t)},\Delta)). \tag{143}$$

We will bound this using Hoeffding's inequality(Lemma 8). To this end, we need upper and lower bounds on δf. We can get this easily from Lemma 4,

$$0 < \delta f(E_u(\underline{\sigma}, \underline{\theta}^*), E_u(\underline{\sigma}, \Delta)) \leq \frac{||\Delta_u||_1^2}{2} \leq 2\gamma^2. \tag{144}$$

Now using Hoeffding inequality,

$$Pr\left(\delta\mathcal{L}_M(\Delta, \underline{\theta}^*) \leq \delta\mathcal{L}(\Delta, \underline{\theta}^*) - \varepsilon_b\right) \leq \exp\left(\frac{M\varepsilon_b^2}{2\gamma^4}\right) \tag{145}$$

So choosing $M = \frac{2\gamma^4}{\varepsilon_b^2}\log(\frac{1}{\delta_b})$ ensures that $\delta\mathcal{L}_M(\Delta, \underline{\theta}^*) > \delta\mathcal{L}(\Delta, \underline{\theta}^*) - \varepsilon_b$ with probability $1 - \delta_b$. Now using the lower bound in Lemma 5 gives us the required lower bound in the lemma. □

Lemma 7. (Closeness of conditional variance)
Let μ and ν close in conditionals as defined in Theorem 1. Let $F : \mathbb{R}^{n-1} \to \mathbb{R}$ be an arbitrary function. Then the conditional variances of this random variable under these distributions are also close in the following sense,

$$\mathop{\mathbb{E}}_{\nu}\left|F(\underline{\sigma}_{\setminus i})\left(\mathop{\mathrm{Var}}_{\mu}[\sigma_i|\underline{\sigma}_{\setminus i}] - \mathop{\mathrm{Var}}_{\nu}[\sigma_i|\underline{\sigma}_{\setminus i}]\right)\right| \leq \frac{4\eta}{\omega_P}\left|\max_{\underline{x}\in\{-1,1\}^{n-1}} F(\underline{x})\right|. \tag{146}$$

Proof. First notice the elementary fact that the TV upper bounds the difference in any expectation value, $|\mathop{\mathbb{E}}_{\underline{\sigma}\sim P} x(\underline{\sigma}) - \mathop{\mathbb{E}}_{\underline{\sigma}\sim Q} x(\underline{\sigma})| \leq 2|P - Q|_{TV}||x||_\infty$.

Now for a fixed partial configuration of the spin $\underline{\sigma}_{\setminus i}$.

$$\begin{aligned}\mathop{\mathrm{Var}}_{\mu}[\sigma_i|\underline{\sigma}_{\setminus i}] - \mathop{\mathrm{Var}}_{\nu}[\sigma_i|\underline{\sigma}_{\setminus i}] &= \mathop{\mathbb{E}}_{\mu}[\sigma_i^2|\underline{\sigma}_{\setminus i}] - \mathop{\mathbb{E}}_{\nu}[\sigma_i^2|\underline{\sigma}_{\setminus i}] \\ &\quad - \left(\mathop{\mathbb{E}}_{\mu}[\sigma_i|\underline{\sigma}_{\setminus i}] - \mathop{\mathbb{E}}_{\nu}[\sigma_i|\underline{\sigma}_{\setminus i}]\right)\left(\mathop{\mathbb{E}}_{\mu}[\sigma_i|\underline{\sigma}_{\setminus i}] + \mathop{\mathbb{E}}_{\nu}[\sigma_i|\underline{\sigma}_{\setminus i}]\right)\end{aligned} \tag{147}$$

The first term vanishes as $\sigma_i^2 = 1$. Let $|\mu(.|\underline{\sigma}_{\setminus i}) - \nu(.|\underline{\sigma}_{V\setminus u})|_{TV} := \delta_i(\underline{\sigma}_{\setminus i})$. Now using the TV upper bound,

$$\left|\mathop{\mathrm{Var}}_{\mu}[\sigma_i|\underline{\sigma}_{\setminus i}] - \mathop{\mathrm{Var}}_{\nu}[\sigma_i|\underline{\sigma}_{\setminus i}]\right| \leq 4\delta_i(\underline{\sigma}_{\setminus i}). \tag{148}$$

This implies that from Theorem 1

$$\mathop{\mathbb{E}}_{\nu}\left|F(\underline{\sigma}_{\setminus i})\left(\mathop{\mathrm{Var}}_{\mu}[\sigma_i|\underline{\sigma}_{\setminus i}] - \mathop{\mathrm{Var}}_{\nu}[\sigma_i|\underline{\sigma}_{\setminus i}]\right)\right| \leq 4\mathop{\mathbb{E}}_{\nu}\left|F(\underline{\sigma}_{\setminus i})\right|\delta_i(\underline{\sigma}_{\setminus i}) \leq \frac{4\eta}{\omega_P}\left|\max_{\underline{x}\in\{-1,1\}^{n-1}} F(\underline{x})\right|. \tag{149}$$

□

Lemma 8 (Hoeffding's Inequality, [8]). Let $X^{(1)}, X^{(2)}, \ldots, X^{(M)}$ be independent and identically distributed random variables such that $a \leq X^{(i)} \leq b$ and $\varphi = \mathbb{E}[X^{(i)}]$ for all $i = 1, 2, \ldots, M$. Define the empirical mean $S_M = \frac{1}{M}\sum_{i=1}^M X^{(i)}$. Then, for any $t > 0$,

$$\mathbb{P}(S_M - \varphi \geq t) \leq \exp\left(-\frac{2Mt^2}{(b-a)^2}\right),$$

and

$$\mathbb{P}(S_M - \varphi \leq -t) \leq \exp\left(-\frac{2Mt^2}{(b-a)^2}\right),$$

and

$$\mathbb{P}(|S_M - \varphi| \leq t) \leq 2\exp\left(-\frac{2Mt^2}{(b-a)^2}\right).$$

B. Structure learning

First we identify the edges in the true d-sparse graph by the following criterion,

$$\hat{E} = \{(u,v) \in [n] \times [n] \;\; | \;\; \max(|\hat{\theta}_{uv}|, |\hat{\theta}_{vu}|) > \alpha/2\} \tag{150}$$

Choose $\varepsilon = \alpha/4$. This in turn implies that $M = \left\lceil 2^{10} \frac{e^{8\gamma}\gamma^4}{\varepsilon^4} \log(\frac{8n}{\delta}) \right\rceil = \left\lceil 2^{26} \frac{e^{8\gamma}\gamma^4}{\alpha^4} \log(\frac{8n}{\delta}) \right\rceil$.

Now the condition assumed in the theorem is $\alpha > 16e^{2\gamma}\sqrt{\frac{(1+\gamma)\eta}{\omega_P}}$. Now the upper bound on error in the pairwise terms from Theorem 3 is $\varepsilon + 4e^{2\gamma}\sqrt{\frac{(1+\gamma)\eta}{\omega_P}}$. The assumed condition lower bound on α then implies that $\varepsilon + 4e^{2\gamma}\sqrt{\frac{(1+\gamma)\eta}{\omega_P}} < \alpha/2$. This implies that if $\theta *_{u,v}$ is zero, then the PL estimate $t\hat{heta}_{u,v}$ is guaranteed to be less than $\alpha/2$ with high probability. On the other hand is $\theta^*_{u,v} \neq 0$, the estimated value will be greater that $\alpha/2$. So from Theorem 3, with probability $1-\delta$, the estimated structure $\hat{E}$ matches the true structure of μ. This proves the structure learning result.

C. Recovering magnetic fields

We will use the following shorthands for vectors $\underline{\theta}_{E_u} = [\theta_{u,v} | (u,v) \in E]$ and $\underline{\sigma}_{E_u} = [\sigma_v | (u,v) \in E]$. The PL estimator for the magnetic field in given by,

$$\hat{\theta}_u = \underset{|\theta_u| \leq h_{max}}{\operatorname{argmin}} \;\; \frac{1}{M}\sum_{t=1}^{M} \log(1+\exp(-2\sigma_u^{(t)}(\langle \underline{\hat{\theta}}_{E_u}, \underline{\sigma}_{\setminus u}^{(t)}\rangle + \theta_u)). \tag{151}$$

The same machinery that we used prove Theorem 3 can be used to show that $\hat{\theta}_u$ is close to θ^*_u.

Define the following loss functions,

$$\mathcal{H}_M(\theta_u) = \frac{1}{M}\sum_{t=1}^{M} \log(1+\exp(-2\sigma_u^{(t)}(\langle \underline{\hat{\theta}}_{E_u}, \underline{\sigma}_{E_u}^{(t)}\rangle + \theta_u)). \tag{152}$$

$$\mathcal{H}(\theta_u) = \underset{\underline{\sigma}\sim\nu}{\mathbb{E}} \log(1+\exp(-2\sigma_u(\langle \underline{\hat{\theta}}_{E_u}, \underline{\sigma}_{E_u}\rangle + \theta_u)). \tag{153}$$

Also define the curvature of this loss,

$$\delta\mathcal{H}(\Delta_u, \theta_u) = \mathcal{H}(\theta_u + \Delta_u) - (\mathcal{H}(\theta_u) + \Delta_u \frac{\partial H(\theta_u)}{\partial \theta_u}) \tag{154}$$

Now using the same argument that was used to derive (112), we can show that

$$\delta\mathcal{H}_M(\Delta_u, \theta^*_u) \leq 2h_{max}\left|\frac{\partial \mathcal{H}_M(\theta^*_u)}{\partial \theta_u}\right|. \tag{155}$$

We will exploit this relation in the same way as done in the proof of Theorem 3.

1. *Upperbound on gradient*

Upperbounding the gradient as in Lemma 3 is slightly trickier in this case due to the fixed variables in the optimization. First define the following sigmoid type function,

$$S(x) := \frac{1}{1+\exp(2x)}. \tag{156}$$

$$\frac{\partial \mathcal{H}(\theta_u^*)}{\partial \theta_u} = -2 \mathop{\mathbb{E}}_{\underline{\sigma}\sim\nu} \sigma_u S\left(\langle \underline{\hat{\theta}}_{E_u}, \underline{\sigma}_{E_u}\rangle + \theta_u^*\right). \tag{157}$$

Now we can add and subtract $2 \mathop{\mathbb{E}}_{\underline{\sigma}\sim\nu} \sigma_u S\left(\langle \underline{\theta}^*_{E_u}, \underline{\sigma}_{E_u}\rangle + \theta_u^*\right)$. This term can be bounded from Lemma 2 and hence we can write,

$$\begin{aligned} |\frac{\partial \mathcal{H}(\theta_u^*)}{\partial \theta_u}| &\leq 2 \mathop{\mathbb{E}}_{\underline{\sigma}\sim\nu} \left| S\left(\langle \underline{\hat{\theta}}_{E_u}, \underline{\sigma}_{E_u}\rangle + \theta_u^*\right) - S\left(\langle \underline{\theta}^*_{E_u}, \underline{\sigma}_{E_u}\rangle + \theta_u^*\right)\right| + \frac{4\eta}{\omega_P}, & (158)\\ &\leq 2|\langle \underline{\hat{\theta}}_{E_u} - \underline{\theta}^*_{E_u}, \underline{\sigma}_{E_u}\rangle| \sup_{x\in[-\gamma,\gamma]} |S'(x)| + \frac{4\eta}{\omega_P}, & (159)\\ &\leq 4||\underline{\hat{\theta}}_{E_u} - \underline{\theta}^*_{E_u}||_1 + \frac{4\eta}{\omega_P}, & (160)\\ &\leq 4d\varepsilon + \frac{4\eta}{\omega_P}. & (161) \end{aligned}$$

In the third line, we have used Holders inequality to bound the inner product and also used the explicit formula for $|S'(x)| = |2S(x)S(-x)| < 2$ to bound the gradient.

In the last line, we have exploited the fact that the estimates of the pairwise terms are only non-zero on the edges in E. This can be assumed as we know the structure of the underlying model. Then fact that the graph as at most degree d allows us to bound the ℓ_1 norm of the error with ℓ_∞ norm, and gaining only a factor of d in the process.

Clearly $-1 \leq \sigma_u S\left(\langle \underline{\hat{\theta}}_{E_u}, \underline{\sigma}_{E_u}\rangle + \theta_u^*\right) \leq 1$. Hence by Hoeffdings inequality (Lemma 8),

$$Pr\left(\left|\frac{\partial \mathcal{H}(\theta_u^*)}{\partial \theta_u} - \frac{\partial \mathcal{H}_M(\theta_u^*)}{\partial \theta_u}\right| \geq \epsilon_a\right) \leq 2\exp(-\frac{M\epsilon_a^2}{2}) \tag{162}$$

Hence with $M \geq \frac{2}{\epsilon_a^2}\log(\frac{4}{\delta})$, we can ensure with probability at least $1-\delta/2$ that

$$\left|\frac{\partial \mathcal{H}_{\mathcal{M}}(\theta_u^*)}{\partial \theta_u}\right| \leq 4d\varepsilon + \frac{4\eta}{\omega_P} + \epsilon_a \tag{163}$$

2. *Lower bound on curvature*

Now the curvature can be bounded using strong convexity arguments as in Lemma 4. For this we first connect δH to δf, with f as defined in (130),

$$\begin{aligned} \delta H(\Delta_u, \theta_u^*) &= \mathop{\mathbb{E}}_{\underline{\sigma}\sim\nu} \left(f(\sigma_u(\langle \underline{\hat{\theta}}_{E_u}, \underline{\sigma}_{E_u}\rangle + \theta_u)) - f(\sigma_u(\langle \underline{\hat{\theta}}_{E_u}, \underline{\sigma}_{E_u}\rangle + \theta_u^*)) - \Delta_u \sigma_u f'(\sigma_u(\langle \underline{\hat{\theta}}_{E_u}, \underline{\sigma}_{E_u}\rangle + \theta_u^*))\right), & (164)\\ &= \mathop{\mathbb{E}}_{\underline{\sigma}\sim\nu} \delta f(\Delta_u \sigma_u, \theta_u^*). & (165) \end{aligned}$$

Hence from the lower bound in Lemma 4,

$$\delta H(\Delta_u, \theta_u^*) \geq \frac{e^{-2h_{max}}}{2} \mathop{\mathbb{E}}_{\underline{\sigma}\sim\nu} (\Delta_u \sigma_u)^2 = \frac{e^{-2h_{max}}}{2} (\Delta_u)^2 \tag{166}$$

Now from the upper bound in Lemma 4, we have $0 < \delta H(\Delta_u, \theta_u^*) \leq \frac{h_{max}^2}{2}$.

We can use these these bounds with Hoeffding's inequality (Lemma 8) to get the following probabilistic upper-bound on the estimated curvature,

$$Pr\left(\delta H_M(\Delta_u, \theta_u^*) \leq \delta H(\Delta_u, \theta_u^*) - \epsilon_b\right) \leq e^{\frac{-8\epsilon_b^2 M}{h_{max}^4}}. \tag{167}$$

Hence ,$M \geq \frac{h_{max}^4}{8\epsilon_b^2} \log(\frac{2}{\delta})$ ensures that the corresponding lower bound holds w.p greater than $1 - \frac{\delta}{2}$.

3. *Magnetic field learning guarantee*

Now using the bounds on gradient and curvature in (155) and using the union bound, we have the following bound that holds with probability $1-\delta$ when M is greater than $\max\left(\frac{h_{max}^4}{8\epsilon_b^2}\log(\frac{2}{\delta}), \frac{2}{\epsilon_a^2}\log(\frac{4}{\delta})\right)$,

$$\frac{e^{-2h_{max}}}{2}\Delta_u^2 \leq \epsilon_b + 2h_{max}\left(4d\varepsilon + \frac{4\eta}{\omega_P} + \epsilon_a\right) \tag{168}$$

Now choosing $\frac{\varepsilon_h^2}{2} = 2e^{2h_{max}}\epsilon_b = 4h_{max}e^{2h_{max}}\epsilon_a$, gives us

$$\Delta_u \leq \varepsilon_h + 4\sqrt{d\varepsilon h_{max}}e^{h_{max}} + 4\sqrt{\frac{\eta h_{max}}{\omega_P}}e^{h_{max}}. \tag{169}$$

Plugging the definition of ε_h in the M values, we see that this can be achieved with $M = \left\lceil \frac{2^7 h_{max}^4 e^{4h_{max}}}{\varepsilon_h^4} \right\rceil$ samples.

---

[1] Nash-Williams, C. S. J. Random walk and electric currents in networks. In *Mathematical Proceedings of the Cambridge Philosophical Society* (1959), vol. 55, Cambridge University Press, pp. 181–194.
[2] Doyle, P. G., and Snell, J. L. *Random walks and electric networks*, vol. 22. American Mathematical Soc., 1984.
[3] Horn, R. A., and Johnson, C. R. *Matrix analysis*. Cambridge university press, 2012.
[4] Kochmański, M., Paszkiewicz, T., and Wolski, S. Curie–weiss magnet—a simple model of phase transition. *European Journal of Physics 34*, 6 (2013), 1555.
[5] Levin, D. A., Luczak, M. J., and Peres, Y. Glauber dynamics for the mean-field ising model: cut-off, critical power law, and metastability. *Probability Theory and Related Fields 146* (2010), 223–265.
[6] Götze, F., Sambale, H., and Sinulis, A. Higher order concentration for functions of weakly dependent random variables. *Electronic Journal of Probability 24*, none (2019), 1 – 19.
[7] Zhou, X. On the fenchel duality between strong convexity and lipschitz continuous gradient. *arXiv preprint arXiv:1803.06573* (2018).
[8] Vershynin, R. *High-dimensional probability: An introduction with applications in data science*, vol. 47. Cambridge university press, 2018.
[9] Negahban, S. N., Ravikumar, P., Wainwright, M. J., and Yu, B. A unified framework for high-dimensional analysis of m-estimators with decomposable regularizers. *Statistical Science 27*, 4 (2012), 538–557.
