# Supplementary material for: Discrete distributions are learnable from metastable samples
Source: Nat Commun. 2026 Jul 23;17:8957. doi: 10.1038/s41467-026-75439-1 (PMC13503862; doi:10.1038/s41467-026-75439-1)
Supplement: Supplementary file 2 [file 41467_2026_75439_MOESM2_ESM.pdf]

nature portfolio

Peer Review File

Discrete distributions are learnable from metastable samples

Corresponding Author: Dr Abhijith Jayakumar

This file contains all reviewer reports in order by version, followed by all author rebuttals in order by version.

Attachments originally included by the reviewers as part of their assessment can be found at the end of this file.

Version 0:

Reviewer comments:

Reviewer #2

(Remarks to the Author)
Systems which exhibit metastability--that is, where the dynamics are stuck in certain regions of the state space for an extended time--can present obstacles for algorithms which attempt to learn the stationary distribution. However, more recent work suggests that modified algorithms or analyses can provide algorithms even for metastable systems.

Many past works consider samples that are either independent, or sequential from a Markov chain, from the stationary distribution. This work asks the question of whether we can learn given (independent) samples that are themselves drawn from a metastable state, e.g., one portion of the state space. The motivation is that for multimodal distributions, sampling can be computationally difficult and it can be unreasonable to assume that we are given such samples.

This is an interesting new variant of the problem that is arguably closer to practice. The key result is that under some reasonable assumptions, pseudo-likelihood (a standard algorithm for learning) can work even in this problem setting. This uses the observation that the single variable conditionals ($\sigma_i|\sigma_{-i}$) for a metastable state are close to that of the distribution.

Key to the result is coming up with the right notion of metastability. The authors define a new notion (eta-strong metastability) useful for learning, which is highly intuitive and shown to be satisfied for some simple systems. The proof is straightforward after making this definition correctly and conjecturing the result. They also compare different notions of metastability.

This is a very nice direction; however, it feels like only a first step. Given that the techniques used are quite simple, it seems that one could hope for much more comprehensive results.

* Generally speaking, systems that exhibit fast mixing are known to be easy to learn; this work considers the opposite setting, showing learning guarantees under a strong metastable assumption. However, it is left open what happens in between these two regimes--the results break down here. A priori, a system might have many modes and exhibit slow mixing, while being hard to extract samples from a metastable state thereof, as there can be a range of different amounts of overlap/connectivity between the modes.

* Unlike iid samples from the distribution itself, the assumption of iid samples from a metastable state feels less motivated. For example, a more natural assumption which is related but different may be that the samples come from a run of the Markov chain that is long enough to locally, but not globally mix. Under this type of assumption, one might expect results that interpolate between the regimes. In this formulation, it's not clear if the metastability is needed or is in fact a red herring.

* The result includes bias, in contrast to other learning guarantees. It is unclear whether this bias is inherent or not. If it is, an impossibility result would complete the story here (again, this would be more convincing under a more "natural" assumption on data acquisition as above).

* Condition 2 is crucial for the current proof; however, it is a strong condition that is not satisfied for many systems of interest. It has exponential dependence on the interaction strength (a L^1/sparsity-like assumption), and is in the older line of work on learning graphical models. However, even systems that exhibit good mixing need not have small L^1 norm, in particular, mean-field type models such as the Sherrington-Kirkpatrick model.

* The relevance of constructing strongly metastable distributions for the Curie-Weiss model with Taylor expansion is unclear, as this approach gives much weaker metastability than simple truncation.

There are several relevant papers that would be good to compare to, which also consider learning for models that do not exhibit fast mixing.

[1] proves learning guarantees SK model at low temperature, which exhibits slow mixing but as a mean-field model does not satisfy the L^1 conditions.

[2] and gives a framework that simultaneously works for discrete and continuous systems, and give guarantees for approximate low-rank Ising models--again not satisfying the L^1 conditions.

[3] considers a continuous setting with score matching, for a kind of p-spin model.

References:

[1] Chandrasekaran, G., & Klivans, A. R. (2025, June). Learning the sherrington-kirkpatrick model even at low temperature. In Proceedings of the 57th Annual ACM Symposium on Theory of Computing (pp. 1774-1784).

[2] Koehler, F., Lee, H., & Vuong, T. D. (2025). Efficiently learning and sampling multimodal distributions with data-based initialization. In Conference on Learning Theory (pp. 3264-3326). PMLR.

[3] Pabbaraju, C., Rohatgi, D., Sevekari, A. P., Lee, H., Moitra, A., & Risteski, A. (2023). Provable benefits of score matching. Advances in Neural Information Processing Systems, 36, 61306-61326.

The paper is clearly written and mathematically sound, but can benefit from a round of copy-editing.

(Remarks on code availability)

Reviewer #3

(Remarks to the Author)
In this manuscript, the authors present a key finding: even when a Markov chain mixes slowly, it is still possible to recover the true stationary distribution model using conditional-likelihood-based methods (such as the pseudo-likelihood), even if the samples are drawn from a metastable distribution that differs drastically from the stationary distribution in global metrics. The topic is interesting and the manuscript is generally well written, but the issues outlined below need to be addressed.

1. Theorem 1's learning guarantee relies exclusively on η-strong metastability. The broad applicability of the result requires establishing a tighter link between this condition and the intuitive η-metastability. Since Proposition 2 shows a potential separation proportional to diam(G), the authors must either provide numerical evidence that typical η-metastability in slow-mixing regimes are practically close to η-strong metastability, and explicitly discuss how the bias O(n\eta) (when \omega_P =1/n) is theoretically overcome by the exponentially small η in large systems, ensuring the bias O(nη) remains negligible for general metastable states.

2. Theorem 4 shows that nonzero coupling terms α must exceed a threshold dependent on the intrinsic bias (α> 16exp(2γ)√(1+γ)η / ω_p) for reliable structure learning. It is essential to discuss the practical impact of this inherent, bias-dependent threshold on structure learning in settings characterized by high sparsity and strongly heterogeneous interaction strengths.

3. The rigorous theoretical guarantees (Theorem 3, 4, 5) focus solely on the binary pairwise Ising model, and the simulations are limited to the fully connected Curie–Weiss model. To better demonstrate the method's effectiveness and support claims of generalizability, the authors should expand the numerical experiments to include non-binary variables (e.g., Potts model) and sparse graph structures, to empirically validate structure learning in realistic settings.

(Remarks on code availability)

Reviewer #4

(Remarks to the Author)
The key point of this paper is to argue that when learning an undirected graphical model from data using pseudolikelihood-style methods, the assumption that the data is generated from the true Gibbs measure/Boltzmann distribution can be weakened to requiring that the data is generated from a metastable state. Here a metastable state is a distribution which is close to stationary under short-time evolution of the Glauber dynamics or similar Markov chain.

- The writing and figures seem generally satisfactory to me.

- The high-level point makes plenty of sense. I have not seen it explicitly pointed out in the literature before so I think it is worth publishing. Given the high-level idea, the results seem very intuitively reasonable.

- Intuitively, the phenomenon that occurs here is related to the non-uniqueness of Gibbs states in statistical physics systems on infinite graphs. (E.g. as in Chapter 6 of the book by Velenik et al https://www.unige.ch/math/folks/velenik/smbook/). For systems on finite graphs, there is a unique Gibbs measure, but in the infinite case there can be several measures with the correct conditional laws and thisw is what is key for pseudolikelihood to work, and also it is related to the existence of metastable states. It would be nice to mention this briefly.

- It may be worth briefly mentioning some other recent work which combines, in a different way, metastability and learning such as https://arxiv.org/abs/2411.09117 (Koehler-Lee-Vuong). The setup is quite different (in their setting, it is crucial that the samples are generated from the true Gibbs measure, so that the distribution can be recovered even if the parameters cannot) but the mathematics related to metastability in the Curie-Weiss model and generalizations is in a similar spirit.

(Remarks on code availability)

Version 1:

Reviewer comments:

Reviewer #2

(Remarks to the Author)
In comparison to the first draft, the authors further motivate their definition of strong metastability by showing robustness under taking mixtures of metastable distributions (for a fixed Markov chain) and under time evolution, and provide further experimental results for a Potts model.

I would like to see more discussion in the paper on when one could expect to be able to draw from an eta-metastable distribution, as in the author response. In particular, the discussion on when the authors' definition of a metastable distribution describes "samples from a Markov chain that has run enough to mix locally" should be included in the paper. Ideally, this would be a formal mathematical statement with proof (under certain conditions, collecting samples from a run of a Markov chain would give close-to-iid samples from a metastable distribution); this would also serve as a reduction from the previously considered setting of Markov chain trajectories.

A priori, the statement "mixing time that is sufficient for local mixing but not global mixing" may hide an assumption on a separation between these mixing times. In this direction, please note the following comment was meant regarding the stationary distribution, not the metastable distribution, and hence was not addressed by the response:
> A priori, a system might have many modes and exhibit slow mixing, while being hard to extract samples from a metastable state thereof, as there can be a range of different amounts of overlap/connectivity between the modes.
The results of the paper seem most useful when eta is very small (e.g., exponentially small in n). This is the case for statistical physics models at low temperature, but there are interesting settings/regimes when this is not the case, as noted above (e.g., only moderately separated modes). As a further example, near the critical temperature, eta might only be polynomially small. It is worth noting limitations of the metastability assumption in these settings.

I believe that the authors' definition of metastability and assumption of iid samples from a metastable distribution is reasonable; however, given their importance in the paper, a more careful discussion situating these assumptions is warranted, and a formal connection with other settings would strengthen the centrality of this definition/assumption.

Minor:

* There is some missing punctuation at the end of theorem statements.

* "To demonstrate learning from metastable samples in the most general setting, we study this learning problem on a Potts-type model" - This statement is a bit misleading because the Potts model considered is highly specific; it would be more appropriate to say that this provides an example for q>2.

(Remarks on code availability)

Reviewer #3

(Remarks to the Author)
The manuscript has been substantially improved, and the theoretical claims are now well supported by the additional numerical results. I have no further comments, and congratulate the authors on this nice work.

(Remarks on code availability)

Reviewer #4

(Remarks to the Author)
I am satisfied with the revision.

(Remarks on code availability)

Version 2:

Reviewer comments:

Reviewer #2

(Remarks to the Author)
In response to my previous review, the authors have added discussions on the metastability assumption, including connections to prior work and open questions, and I recommend acceptance.

(Remarks on code availability)

Open Access This Peer Review File is licensed under a Creative Commons Attribution 4.0 International License, which permits use, sharing, adaptation, distribution and reproduction in any medium or format, as long as you give appropriate credit to the original author(s) and the source, provide a link to the Creative Commons license, and indicate if changes were made.
In cases where reviewers are anonymous, credit should be given to 'Anonymous Referee' and the source.
The images or other third party material in this Peer Review File are included in the article's Creative Commons license, unless indicated otherwise in a credit line to the material. If material is not included in the article's Creative Commons license and your intended use is not permitted by statutory regulation or exceeds the permitted use, you will need to obtain permission directly from the copyright holder.
To view a copy of this license, visit https://creativecommons.org/licenses/by/4.0/

Dear *Nature Communications* Editors,

We would like to thank all the Reviewers for a careful and thorough review of our manuscript and their useful remarks that allowed us to improve the presentation. We have significantly revised the manuscript following their recommendations. We would like to highlight two major additions to the paper. In the new Section II B, we show that the property of strong metastability is robust to taking a mixture of distributions and also to evolving by the Markov chain. In the new Section V C, we demonstrate our learning results on a spin glass model with higher-order interactions and a Potts alphabet.

Please note that for Reviewers' convenience, major additions have been highlighted in blue and deletions in red in the text.

Below we provide a detailed response to the specific comments made by the Reviewers.

Yours sincerely,

The Authors

Response to the specific comments made by Reviewers.

Reviewer #2.

Systems which exhibit metastability–that is, where the dynamics are stuck in certain regions of the state space for an extended time–can present obstacles for algorithms which attempt to learn the stationary distribution. However, more recent work suggests that modified algorithms or analyses can provide algorithms even for metastable systems.

Many past works consider samples that are either independent, or sequential from a Markov chain, from the stationary distribution. This work asks the question of whether we can learn given (independent) samples that are themselves drawn from a metastable state, e.g., one portion of the state space. The motivation is that for multimodal distributions, sampling can be computationally difficult and it can be unreasonable to assume that we are given such samples.

This is an interesting new variant of the problem that is arguably closer to practice. The key result is that under some reasonable assumptions, pseudo-likelihood (a standard algorithm for learning) can work even in this problem setting. This uses the observation that the single variable conditionals $(\sigma_i|\sigma_{-i})$ *for a metastable state are close to that of the distribution.*

Key to the result is coming up with the right notion of metastability. The authors define a new notion (eta-strong metastability) useful for learning, which is highly intuitive and shown to be satisfied for some simple systems. The proof is straightforward after making this definition correctly and conjecturing the result. They also compare different notions of metastability.

This is a very nice direction; however, it feels like only a first step. Given that the techniques used are quite simple, it seems that one could hope for much more comprehensive results.

Answer: We are thankful to the Reviewer for their positive assessement of our research approach, and for their remarks that allowed us to improve the presentation.

** Generally speaking, systems that exhibit fast mixing are known to be easy to learn; this work considers the opposite setting, showing learning guarantees under a strong metastable assumption. However, it is left open what happens in between these two regimes–the results break down here. A priori, a system might have many modes and exhibit slow mixing, while being hard to extract samples from a metastable state thereof, as there can be a range of different amounts of overlap/connectivity between the modes.*

Answer:
We are grateful to the Reviewer for this remark, as this is an important point. We would like to point out that the definition of strong metastability is compatible with the metastable distribution having support on multiple modes. Specifically, given a collection of metastable distributions ν_i each of which are η strongly metastable, any mixture of these distribution will also be η- strongly metastable. We have included the derivation of this propoerty in a new section (II B). Also, we have included new numerical experiments in Section V, where we demonstrate learning from a spin-glass model, which is expected to have multiple local minima where the dynamic can get stuck. Hence, our framework extends to the case of multiple modes in a straightforward way.

Unlike iid samples from the distribution itself, the assumption of iid samples from a metastable state feels less motivated. For example, a more natural assumption which is related but different may be that the samples come from a run of the Markov chain that is long enough to locally, but not globally mix. Under this type of assumption, one might expect results that interpolate between the regimes. In this formulation, it's not clear if the metastability is needed or is in fact a red herring.

Answer:
We view our definition of metastable distribution as a distribution describing samples from a Markov chain that has run enough to mix locally. We agree that this may appear as unnatural to a degree, because the distribution is always moving towards the stationary state, albeit very slowly. Despite this, we have three arguments for why this definition is indeed the right one for the study of learning algorithms, both in the theoretical and practical settings.

- In practice, the drift from a metastable distribution is almost negligible. This what makes metastability an adversarial property from the perspective of sampling. For a practical illustration, consider the setup for the experiment on a Potts spin glass that we have added in the new Section V C. We see that the dynamics is restarted only 16 times while up to $M = 10^5$ samples are collected. But we see that the drift induced by this does not degrade the learning results.
- On the theory side, we have also added a new result in section II B (Propostion 2) that bounds how strong metastability changes under the action of Markov chain. Specifically, we show that an $\eta-$strongly metastable state remains at least $(1+2t)\eta$-strongly metastable when evolved by the Markov chain for t steps. Since interesting cases of metastable states correspond to exponentially small η in the number of variables, this shows that once the distribution described by the chain becomes strongly metastable, subsequent samples obtained from running Markov dynamics also come from strongly metastable distributions for sufficiently long times.
- Collection of an i.i.d sample from a metastable stable state can be done in the following way: assume that, as the Reviewer suggested, there exits a mixing time that is sufficient for local, but not global, mixing. To collect i.i.d samples from a metastable distribution, we can restart the dynamics from an initial state and collect single sample after the dynamics has relaxed to a metastable state. By repeating this procedure, and making sure to collect a sample at the same time at each run, we can ensure that an i.i.d sample is collected from the metastable distribution. This way of collecting samples is possible if the learner has the ability to completely reset the state of the system after recording the sample.

** The result includes bias, in contrast to other learning guarantees. It is unclear whether this bias is inherent or not. If it is, an impossibility result would complete the story here (again, this would be more convincing under a more "natural" assumption on data acquisition as above).*

Answer:
The bias in the learning seems to be inherent, at least if the learning goes through the conditionals. The conditionals of the metastable distribution agrees with the conditionals of the true distribution only approximately. Since there is a strict bijection between the conditionals and the full distribution, any procedure that learns via conditionals must introduce a bias. This argument does not rule out learning via methods that bypass the conditionals completely. Proving such an impossibility result would be remarkable, but we do not believe that the tools to show such a strong algorithmic result currently exist in learning theory.

** * Condition 2 is crucial for the current proof; however, it is a strong condition that is not satisfied for many systems of interest. It has exponential dependence on the interaction strength (a L^1/sparsity-like assumption), and is in the older line of work on learning graphical models. However, even systems that exhibit good mixing need not have small L^1 norm, in particular, mean-field type models such as the Sherrington-Kirkpatrick model.*

Answer: Condition 2 (the ℓ_1 width) cannot be avoided unless a concession is made elsewhere, either on the class of models considered or on the strictness of the learning guarantees.

- The information theoretic lower-bound on learning undirected graphical models depends on the ℓ_1 width of the model [5].
- The recent learning results on SK models are probabilistic statements over the class of models [1], and hence are not the worst-case statements. Also, the sample complexity in these results scale at least as $\exp(\beta\sqrt{\log(n)})$, where n is the number of variables. In comparison, the parameter learning results in our work apply to all models in the Ising class. Also, the sample complexity results we have are "high-dimensional", i.e., the required number of samples scale as $O(\log(n))$. It may be interesting to consider if the techniques in the recent SK model results extend to the case of metastable distributions for providing typical-case guarantees. However, as these techniques are not widely applicable, we think this is best left for future work.

The relevance of constructing strongly metastable distributions for the Curie-Weiss model with Taylor expansion is unclear, as this approach gives much weaker metastability than simple truncation.

The motivation for considering this comes from statistical physics, where metastability is characterized by expanding the free energy to low orders in the order parameter (m), à la Ginzburg-Landau. Our aim here is to show explicitly that the metastable distributions constructed in this way are included in our definition of strong metastability. Given the interdisciplinary nature of the journal, we think it is important to make this connection. We have added additional text in the paper explaining this motivation. We thank the Reviewer for pointing out that this piece looks unmotivated.

There are several relevant papers that would be good to compare to, which also consider learning for models that do not exhibit fast mixing.
[1] proves learning guarantees SK model at low temperature, which exhibits slow mixing but as a mean-field model does not satisfy the L_1 conditions.
[2] and gives a framework that simultaneously works for discrete and continuous systems, and give guarantees for approximate low-rank Ising models–again not satisfying the L_1 conditions.
[3] considers a continuous setting with score matching, for a kind of p-spin model.
References:
[1] Chandrasekaran, G., & Klivans, A. R. (2025, June). Learning the sherrington-kirkpatrick model even at low temperature. In Proceedings of the 57th Annual ACM Symposium on Theory of Computing (pp. 1774-1784).
[2] Koehler, F., Lee, H., & Vuong, T. D. (2025). Efficiently learning and sampling multimodal distributions with data-based initialization. In Conference on Learning Theory (pp. 3264-3326). PMLR.
[3] Pabbaraju, C., Rohatgi, D., Sevekari, A. P., Lee, H., Moitra, A., & Risteski, A. (2023). Provable benefits of score matching. Advances in Neural Information Processing Systems, 36, 61306-61326.

Answer: We thank the Reviewer for these references and we have included a discussion in our paper clarifying the relationship between these works in Section VI.
Below we address each of them.

- **Chandrasekaran et.al. (2025)** [1]: As mentioned in our reply above, the setting here is not as general as studied by us.
- **Koehler et.al (2025)** [3]: This paper is also an interesting work that considers specific classes of models. Specifically, these require that J has a low rank property. We do not think this is directly relevant to our work, as we are interested in worst case learning guarantees over more general class of models. Also, earlier work from a subset of the same authors give efficient algorithms for this class of models [2], which makes them "easy" models from the inference perspective. In contrast, we are interested in learning in the opposing setting, models where inference is hard and hence metastable distributions are all one can hope to have access to.
- **Pabbaraju et.al. 2023** [4] : We agree that a similar result would be interesting in the setting of continuous variables. However we would like to point out that that the learning guarantees in [4] are asymptotic. So the state of learning results for continuous variable models with score matching (with i.i.d samples from the true distributions) is not as developed as the discrete case. We think these questions are best addressed in a separate work as the techniques required to prove such results will be substantially different.

The paper is clearly written and mathematically sound, but can benefit from a round of copy-editing.

We thank the reviewer for acknowledging the clarity of our work. We have corrected the typos in the the revised version.

Reviewer #3.

In this manuscript, the authors present a key finding: even when a Markov chain mixes slowly, it is still possible to recover the true stationary distribution model using conditional-likelihood-based methods (such as the pseudo-likelihood), even if the samples are drawn from a metastable distribution that differs drastically from the stationary distribution in global metrics. The topic is interesting and the manuscript is generally well written, but the issues outlined below need to be addressed.

Answer: We thank the Reviewer for their time in reviewing our paper, providing useful remarks that helped us improve the manuscript, and for finding the topic interesting.

1. Theorem 1's learning guarantee relies exclusively on η-strong metastability. The broad applicability of the result requires establishing a tighter link between this condition and the intuitive η-metastability. Since Proposition 2 shows a potential separation proportional to diam(G), the authors must either provide numerical evidence that typical η-metastability in slow-mixing regimes are practically close to η-strong metastability, and explicitly discuss how the bias $O(n\eta)$ (when $\omega_P = 1/n$) is theoretically overcome by the exponentially small η in large systems, ensuring the bias $O(n\eta)$ remains negligible for general metastable states.

Answer: Strong metastability is necessary for learning from conditionals. However, it appears that in several natural settings, we have shown that the notion of more intuitive (weak) and strong metastability are identical. For instance, in the paper we have shown two instances:

- **Low-conductance constructions:** These correspond to the notion of a metastable distribution as being stuck in a region of the state space that is bottle-necked. As shown in the paper, these are η-strongly metastable states, with η relating precisely to the degree of bottlenecking experienced by these distributions. From the theory of Markov chains, we know that the conductance is related inversley to the mixing time, so for chains that mix slowly ($t_{mix} \sim exp(n)$), the η here will be exponentially small and O($n\eta$) bias will be negligible.
- **Free energy minima Curie-Weiss:** The states described in Section V-I correspond to expansions around the mimia of the free-energy, as studied in statistical physics. As our numerical experiments in Fig 2 show, these also correspond to strongly metastable distributions. Here as well, by using a hihger order expansion for the free energy or by exactly truncating it around a minima, we can construct metastable states such that $n\eta$ vanishes with the size of the system.
- In the revised version, we have included **a new numerical evidence (Section V C) that we are able to reconstruct spin glass models with Potts variables from metastable states** that further confirms that strong metastability is satisfied by these models.

Overall, there is good evidence that the strong metastability condition is satisfied for natural metastable states. Our example showing the separation between the two types of metastability is a pathological construction: it represents a metastable state which is highly de-localized in the state space.

** 2. Theorem 4 shows that nonzero coupling terms α must exceed a threshold dependent on the intrinsic bias ($\alpha > 16exp(2\gamma)\sqrt{(1+\gamma)}\eta/\omega_p$) for reliable structure learning. It is essential to discuss the practical impact of this inherent, bias-dependent threshold on structure learning in settings characterized by high sparsity and strongly heterogeneous interaction strengths.*

Answer: We thank the Reviewer for their remark regarding this point. Structure learning works by thresholding, i.e., by declaring that the learned edges below a chosen threshold is spurious. The Reviewer is correct that because of the bias in the learning, very small couplings may get swamped by the bias. However, as η is expected to decrease sharply with the size of the of the system, in most practical cases we do not expect the bias to play a significant role. Also in the case of heterogeneous interactions, even if we are in a setting where the bias overwhelms the smallest terms, the larger terms in the energy will be correctly identified by the algorithm.

The new results in Section V, include a structure learning experiment on random hyper graphs. We have also added text below Theorem 4 to emphasize this point.

** 3. The rigorous theoretical guarantees (Theorem 3, 4, 5) focus solely on the binary pairwise Ising model, and the simulations are limited to the fully connected Curie–Weiss model. To better demonstrate the method's effectiveness and support claims of generalizability, the authors should expand the numerical experiments to include non-binary variables (e.g., Potts model) and sparse graph structures, to empirically validate structure learning in realistic settings.*

Answer: We thank the Reviewer for this suggestion. To demonstrate our methods in a more general setting, we have included learning results from a spin-glass model with has both higher order interactions and a higher alphabet size (three state Potts, $q = 3$). We show that the metastable samples from this model allow for parameter and structure learning.

Reviewer #4.

The key point of this paper is to argue that when learning an undirected graphical model from data using pseudolikelihood-style methods, the assumption that the data is generated from the true Gibbs measure/Boltzmann distribution can be weakened to requiring that the data is generated from a metastable state. Here a metastable state is a distribution which is close to stationary under short-time evolution of the Glauber dynamics or similar Markov chain.
- The writing and figures seem generally satisfactory to me.
- The high-level point makes plenty of sense. I have not seen it explicitly pointed out in the literature before so I think it is worth publishing. Given the high-level idea, the results seem very intuitively reasonable.

Answer: We thank the Reviewer for pointing out the novelty of our work.

- Intuitively, the phenomenon that occurs here is related to the non-uniqueness of Gibbs states in statistical physics systems on infinite graphs. (E.g. as in Chapter 6 of the book by Velenik et al https://www.unige.ch/math/folks/velenik/smbook/). For systems on finite graphs, there is a unique Gibbs measure, but in the infinite case there can be several measures with the correct conditional laws and thisw is what is key for pseudolikelihood to work, and also it is related to the existence of metastable states. It would be nice to mention this briefly.

Answer: We thank the Reviewer for pointing this out as this is not something we had thought of before. Yes, at least a subset of the metastable distributions that we consider are related to the measures defined by the DLR conditions in this reference. However, the strong metastability condition only implies that the conditionals match when averaged over the metastable distribution (as shown in Theorem 1). This seems to be a weaker notion than what is defined by DLR. So we don't believe the correspondence is one-to-one. Moreover, our results hold for the finite system sizes. For completeness, we have included this reference and a comment on the relationship between these notions in the conclusion of the paper.

- It may be worth briefly mentioning some other recent work which combines, in a different way, metastability and learning such as https://arxiv.org/abs/2411.09117 (Koehler-Lee-Vuong). The setup is quite different (in their setting, it is crucial that the samples are generated from the true Gibbs measure, so that the distribution can be recovered even if the parameters cannot) but the mathematics related to metastability in the Curie-Weiss model and generalizations is in a similar spirit.

Answer: Thanks to the referee for pointing this out. We agree that this is a valuable reference to include as it deals with an different, but nonetheless interesting learning problem. We have added some text in the conclusion referring to this paper.

References

[1] Chandrasekaran, G., and Klivans, A. R. Learning the sherrington-kirkpatrick model even at low temperature. In *Proceedings of the 57th Annual ACM Symposium on Theory of Computing* (2025), pp. 1774–1784.

[2] Koehler, F., Lee, H., and Risteski, A. Sampling approximately low-rank ising models: Mcmc meets variational methods. In *Conference on Learning Theory* (2022), PMLR, pp. 4945–4988.

[3] Koehler, F., Lee, H., and Vuong, T.-D. Efficiently learning and sampling multimodal distributions with data-based initialization. *arXiv preprint arXiv:2411.09117* (2024).

[4] Pabbaraju, C., Rohatgi, D., Sevekari, A. P., Lee, H., Moitra, A., and Risteski, A. Provable benefits of score matching. *Advances in Neural Information Processing Systems 36* (2023), 61306–61326.

[5] Santhanam, N. P., and Wainwright, M. J. Information-theoretic limits of selecting binary graphical models in high dimensions. *IEEE Transactions on Information Theory 58*, 7 (2012), 4117–4134.

Dear *Nature Communications* Editors,

We would like thank the reviewers for the quick turn around time on this round of revision. We have included a revised version of the manuscript with added text in the Discussion section. The new text is highlighted in blue.

The Authors

Response to the specific comments made by Reviewers.

Reviewer #2.

In comparison to the first draft, the authors further motivate their definition of strong metastability by showing robustness under taking mixtures of metastable distributions (for a fixed Markov chain) and under time evolution, and provide further experimental results for a Potts model.
I would like to see more discussion in the paper on when one could expect to be able to draw from an eta-metastable distribution, as in the author response. In particular, the discussion on when the authors' definition of a metastable distribution describes "samples from a Markov chain that has run enough to mix locally" should be included in the paper. Ideally, this would be a formal mathematical statement with proof (under certain conditions, collecting samples from a run of a Markov chain would give close-to-iid samples from a metastable distribution); this would also serve as a reduction from the previously considered setting of Markov chain trajectories.A priori, the statement "mixing time that is sufficient for local mixing but not global mixing" may hide an assumption on a separation between these mixing times. In this direction, please note the following comment was meant regarding the stationary distribution, not the metastable distribution, and hence was not addressed by the response: ¿ A priori, a system might have many modes and exhibit slow mixing, while being hard to extract samples from a metastable state thereof, as there can be a range of different amounts of overlap/connectivity between the modes. The results of the paper seem most useful when eta is very small (e.g., exponentially small in n). This is the case for statistical physics models at low temperature, but there are interesting settings/regimes when this is not the case, as noted above (e.g., only moderately separated modes). As a further example, near the critical temperature, eta might only be polynomially small. It is worth noting limitations of the metastability assumption in these settings.

Answer: We are grateful to the reviewer for the remarks, which helped us improve the presentation further. In response to the comment above, we have added a detailed discussion, based on our previous answer, on how one can sample from a strongly metastable state. We also connect our definitions of metastability to the works of Liu et al. [2] and Alman et al. [1], where questions of local mixing and sampling are addressed in a more rigorous setting. This addition appears in the *Discussion* section of the paper. There, we also emphasize the difference between our setting and the previous works on learning from Markov chain trajectories.
We agree that our results are most useful when η is small. However, examining the bias in the learning guarantees shows that η need not be exponentially small. Even states with $\eta = O(1/n^2)$ can yield nontrivial learning guarantees for Glauber dynamics in large systems.
We do not view large overlaps between modes as a significant drawback in this context. Such overlaps imply that the chain can transition easily between different modes of the stationary distribution, and the appropriate value of η to consider is that associated with the larger region encompassing these modes. In such cases, the most relevant question is whether the distribution of the chain at time t becomes close, in a reasonable amount of time, to the restriction of the stationary distribution to a low-conductance set. Answering this question remains an outstanding open problem with implications beyond graphical model learning; see Problem 1.23 in Liu et al. [2].

I believe that the authors' definition of metastability and assumption of iid samples from a metastable distribution is reasonable; however, given their importance in the paper, a more careful discussion situating these assumptions is warranted, and a formal connection with other settings would strengthen the centrality of this definition/assumption.

Answer: We are glad that the reviewer thinks that our definitions form a reasonable model for metastability. We believe the updated discussion will convey this sense to the reader, while leaving the independent task of developing a corresponding theory of sampling from states as a fruitful future direction.

** There is some missing punctuation at the end of theorem statements.*
** "To demonstrate learning from metastable samples in the most general setting, we study this learning problem on a Potts-type model" - This statement is a bit misleading because the Potts model considered is highly specific; it would be more appropriate to say that this provides an example for $q > 2$.*

Answer: We have added missing punctuation parks at the end of theorems. Also, we have removed all instances where we called the model with $q > 2$ as Potts-type.

References

[1] Alman, J., Nadimpalli, S., Patel, S., and Servedio, R. A. Dnf learning via locally mixing random walks. In *Proceedings of the 57th Annual ACM Symposium on Theory of Computing* (2025), pp. 2055–2061.

[2] Liu, K., Mohanty, S., Raghavendra, P., Rajaraman, A., and Wu, D. X. Locally stationary distributions: A framework for analyzing slow-mixing markov chains. In *2024 IEEE 65th Annual Symposium on Foundations of Computer Science (FOCS)* (2024), IEEE, pp. 203–215.

In this manuscript, the authors present a key finding: even when a Markov chain mixes slowly, it is still possible to recover the true stationary distribution model using conditional-likelihood-based methods (such as the pseudo-likelihood), even if the samples are drawn from a metastable distribution that differs drastically from the stationary distribution in global metrics. The topic is interesting and the manuscript is generally well written, but the issues outlined below need to be addressed.

1. Theorem 1's learning guarantee relies exclusively on η-strong metastability. The broad applicability of the result requires establishing a tighter link between this condition and the intuitive η-metastability. Since Proposition 2 shows a potential separation proportional to diam(G), the authors must either provide numerical evidence that typical η-metastability in slow-mixing regimes are practically close to η-strong metastability, and explicitly discuss how the bias O(n\eta) (when \omega_P =1/n) is theoretically overcome by the exponentially small η in large systems, ensuring the bias O(nη) remains negligible for general metastable states.

2. Theorem 4 shows that nonzero coupling terms α must exceed a threshold dependent on the intrinsic bias ($\alpha > 16e^{2\gamma}\sqrt{\frac{(1+\gamma)\eta}{\omega_P}}$) for reliable structure learning. It is essential to discuss the practical impact of this inherent, bias-dependent threshold on structure learning in settings characterized by high sparsity and strongly heterogeneous interaction strengths

3. The rigorous theoretical guarantees (Theorem 3, 4, 5) focus solely on the binary pairwise Ising model, and the simulations are limited to the fully connected Curie–Weiss model. To better demonstrate the method's effectiveness and support claims of generalizability, the authors should expand the numerical experiments to include non-binary variables (e.g., Potts model) and sparse graph structures, to empirically validate structure learning in realistic settings.
